# Comparison of blood pool and myocardial 3D printing in the diagnosis of types of congenital heart disease

**DOI:** 10.1038/s41598-022-11294-6

**Published:** 2022-05-03

**Authors:** Jixiang Liang, Xin Zhao, Guangyu Pan, Gen Zhang, Dianjiang Zhao, Jianping Xu, Dianyuan Li, Bingheng Lu

**Affiliations:** 1 The State Key Laboratory for Manufacturing Systems Engineering, Xi’an Jiaotong University, No. 99 Yan Cheung Road, Xi’an, Shannxi China; 2 Institute of 3D Printing, Beijing City University, Beijing, China; 3 Department of Cardiovascular Surgery, Peking University International Hospital, Beijing, China; 4 Department of Cardiovascular Surgery, The Affiliated Traditional Chinese Medicine Hospital of Southwest Medical University, Luzhou, Sichuan China; 5 Department of Radiology, Peking University International Hospital, Beijing, China; 6 Department of Cardiovascular Surgery, The Affiliated Suzhou Hospital of Nanjing Medical University, Suzhou Municipal Hospital, Gusu School, Nanjing Medical University, 26 Daoqian Street, Gusu District, Suzhou, Jiangsu China

**Keywords:** Cardiology, Experimental models of disease

## Abstract

The study aimed to evaluate the effectiveness of blood pool and myocardial models made by stereolithography in the diagnosis of different types of congenital heart disease (CHD). Two modeling methods were applied in the diagnosis of 8 cases, and two control groups consisting of experts and students diagnosed the cases using echocardiography with computed tomography, blood pool models, and myocardial models. The importance, suitability, and simulation degree of different models were analyzed. The average diagnostic rate before and after 3D printing was used was 88.75% and 95.9% (*P* = 0.001) in the expert group and 60% and 91.6% (*P* = 0.000) in the student group, respectively. 3D printing was considered to be more important for the diagnosis of complex CHDs (very important; average, 87.8%) than simple CHDs (very important; average, 30.8%) (*P* = 0.000). Myocardial models were considered most realistic regarding the structure of the heart (average, 92.5%). In cases of congenital corrected transposition of great arteries, Williams syndrome, coronary artery fistula, tetralogy of Fallot, patent ductus arteriosus, and coarctation of the aorta, blood pool models were considered more effective (average, 92.1%), while in cases of double outlet right ventricle and ventricular septal defect, myocardial models were considered optimal (average, 80%).

## Introduction

The presentation of the cardiac structure is of great significance in the diagnosis and treatment of structural, valvular, and congenital heart diseases (CHDs)^1–4^. 3D printing can provide good insight into the 3D anatomy^5,6^, and it has been extensively used in surgical planning and simulation, medical education, interventional procedures, and research for device innovation^6–8^. Fused deposition modeling (FDM), selective laser sintering (SLS), stereolithography (SLA), and material jetting are the most frequently reported 3D printing technologies in cardiovascular medicine^1,9,10^. In addition, to obtain a better sensation of the heart to simulate an operation, vacuum casting technology has been used for the creation of super-flexible heart model^11^. Each 3D printing method and material has advantages and disadvantages^10^. However, in practical applications, the effectiveness, cost, and availability are important factors that restrict the large-scale application of 3D printing in CHD diagnosis^12^. Concerning the techniques used, SLA is a widely used, relatively low-cost, high-precision, and high-speed^13,14^ technique for the fabrication of heart models used in the diagnosis of CHDs^15,16^. In order to get different virtual models, blood pool modeling and myocardial modeling are the common 3D reconstruction methods of cardiac CT or MRI^4^. The segmentation process of appropriate regions of interest can be both automated and manual or, more frequently, semi-automated, combining an initial step of automated segmentation followed by manual corrections^1^. These segmentation and modeling processes have been proved to be effective for 3D printing. Among existing rigid 3D printed heart models, blood pool models^17,18^, myocardial models^7,11^, and their combination^4^ have been used for diagnosis in different studies. Matthew Lee et al^19^ evaluated the feasibility of using rigid blood pool 3D-printed models in cases of coronary artery anomalies. Jiajun Xu et al^15^ assessed the application of blood pool 3D printing in preoperative planning for the treatment of anomalous pulmonary venous connection (APVC) and investigated the roles of 3D-printed blood pool models using SLA technology in presurgical planning for the treatment of complex CHDs with total anomalous pulmonary venous connection (TAPVC), complete transposition of the great arteries (cTGA), patent ductus arteriosus (PDA), ventricular septal defect (VSD), and atrial septal defect (ASD)^18^. However, the existing studies have mainly focused on the feasibly of application in one or more cases, and a comparison of the applicability of blood pool and myocardial 3D printing in different CHDs is lacking. In practical application, which one is more effective for the diagnosis of different types of CHD is unknown. Here, we applied different printing methods in the diagnosis of different CHDs in a retrospective study. Blood pool models and myocardial models were 3D printed and used for the diagnosis of types of CHD. The advantages and disadvantages of the different methods were compared. Through the diagnosis and statistical analysis of typical cases, the improvement in the diagnostic results, satisfaction, accuracy, necessity, and personal preferences of the 3D-printed models in the diagnosis of different CHDs were assessed. This study is of great significance in selecting the type of rigid 3D-printed model for use in the diagnosis of various CHDs.

## Materials and methods

### Study design

This study’s protocol was reviewed and approved by the Medical Ethics Committee of Peking University International Hospital, all methods were carried out in accordance with relevant guidelines and regulations. All the participants signed the informed consent, if the patients are under 18, the informed consent was signed by their parent or legal guardian. To evaluate the effectiveness of different types of 3D prints in the diagnosis of types of CHD, several cases, including cases of complex and simple CHDs, were randomly selected for the study. Rigid blood pool and myocardial models were applied in each case. Control groups with experienced doctors and students diagnosed the disease using echocardiography with computed tomography (CT), blood pool models, and myocardial models. The final results were subject to the consensus of the experts and the results of intraoperative exploration. Doctors or engineers involved in data collection, modeling, 3D printing, and experts agreeing on the final diagnosis did not participate in the subsequent questionnaire survey to ensure respondents are exposed to the data for the first time. In this study, 3D printing was only used for diagnostic statistics and did not interfere with surgical decision-making.

### Case selection

All cases initially diagnosed with CHD by echocardiography and CT in our hospital between January 2020 and December 2020 were included in the study. The cases selected met the following criteria: (1) features consistent with the diagnosis of CHD; and (2) nonemergent situation. To verify the effectiveness of the method, all selected cases were classified by disease subtype. One case from each subtype was randomly selected for detailed comparison.

### Image acquisition, processing, and 3D printing

**Figure 1:**
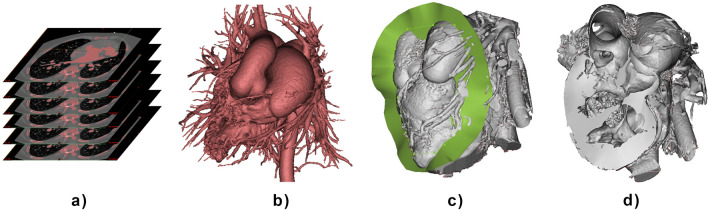
Process of segmentation and modeling. (**a**) Segmentation of CT images. (**b**) Blood pool model. (**c**) Cutting of the myocardial model. (**d**) Myocardial model cut with a flexible surface.

CT was performed with a Siemens SOMATOM Definition Flash Dual-source CT scanner (Siemens Healthcare, Erlangen, Germany). Plain and enhanced CT scans were acquired in turn. The scan was centered on the precordial area, and the scanning range was from 10 to 15 mm below the trachea to the diaphragm of the heart. The data were recorded in Digital Imaging and Communications in Medicine (DICOM) format and imported into Mimics Innovation Suite 19.0 (Materialise HQ, Leuven, Belgium) for processing. To show the complete intracardiac structure, the blood pool area consisting of the aorta, pulmonary artery, atria, ventricles, and superior and inferior vena cavae was selected by the "threshold" method as the region of interest (Fig. 1a) and used for model generation (Fig. 1b). The myocardial model was cut with a flexible surface displaying the anatomical structure of the heart at multiple angles under the guidance of a surgeon familiar with the case (Fig. 1c,d). The software was operated by engineers with more than three years of experience with the assistance of cardiac surgeons with more than five years of experience. To verify the feasibility of 3D reconstruction by CT, blood pool modeling and myocardial modeling were carried out for all collected cases. For the cases selected for detailed comparisons, the blood pool model (Fig. 1b) and the myocardial model cut with a flexible surface (Fig. 1d) were exported for 3D printing.

The final volume meshes of the blood pool and myocardial models were exported as .stl (stereolithography) files. The models were repaired and hollowed using an inward offset; then, a scaffold was added, and the models were sliced in Magics (Materialise HQ, Leuven, Belgium). The models were printed using SLA equipment (Shaanxi Hengtong Intelligent Machine Co., Ltd., Shannxi, China) with rigid white resin.

### Qualitative assessment of clinical value

To assess the clinical value of 3D-printed models of the blood pool and myocardium, a self-designed survey was conducted in two control groups: one consisting of 40 experts (20 cardiac surgeons and 20 sonographers all with more than 10 years of working experience) and the other consisting of 40 students majoring in cardiac surgery and ultrasound (third-year postgraduates). Each participant had access to the CT dataset (original layer by layer sliced images) and echocardiography as well as blood pool and myocardial 3D printing and made independent diagnoses in each case. The interval between each diagnosis was one week to reduce the influence of previous assessments. The diagnostic results using different methods were recorded and compared with the correct diagnosis.

### Statistical analysis

The statistical data were grouped and compared according to several dimensions: traditional (using CT and echocardiography) and new diagnostic methods (using 3D printing), blood pool 3D printing and myocardial 3D printing, expert group and the student group, surgeons and sonographers, complex CHDs and simple CHDs. A series of data analyses were performed to explore the adaptability of different types of 3D printing to individual kinds of CHD. Significant differences in the survey results between the two groups were identified using chi-square tests and Kruskal–Wallis tests according to the change in the value. Statistical analyses were performed using SPSS 21.0 (IBM Corporation, Armonk, NY, USA).

## Results

Forty-five cases of 8 CHD subtypes were collected. All of them (100%, n = 45) have been successfully modeled, blood pool models and myocardium models were generated. One case for each of the 8 subtypes was randomly selected to draw a specific comparison. The diseases included congenital corrected transposition of the great arteries (ccTGA), double outlet right ventricle (DORV), Williams syndrome (WS), coronary artery fistula (CAF), tetralogy of Fallot (TOF), PDA, coarctation of the aorta (CoA), and VSD.

### Modeling and 3D printing

**Figure 2:**
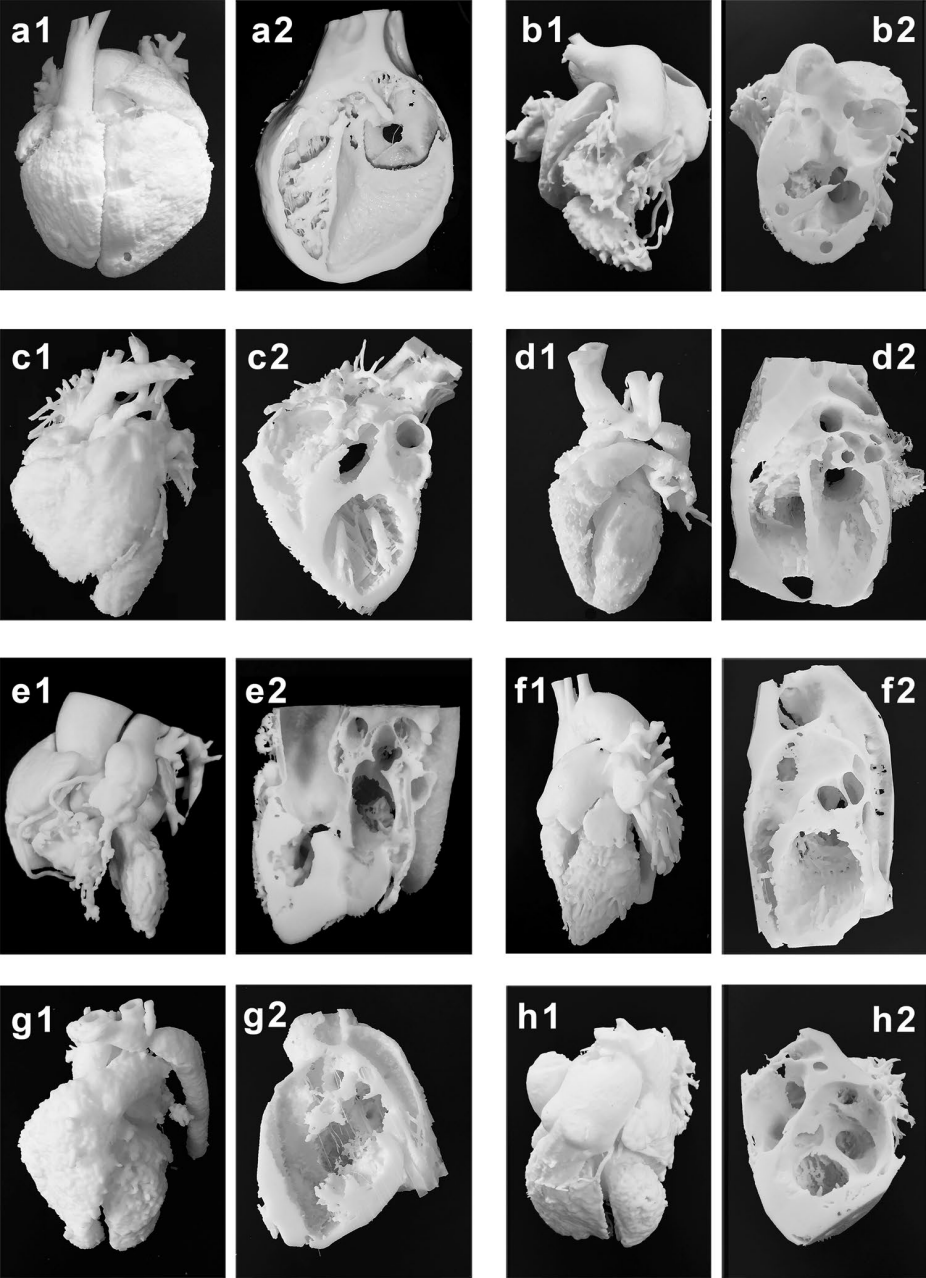
Results of 3D printing of the blood pool and myocardium for eight typical cases. (**a1,a2**) Case 1: ccTGA. (**b1,b2**) Case 2: DORV. (**c1,c2**) Case 3: WS. (**d1,d2**) Case 4: CAF. (**e1,e2**) Case 5: TOF. (**f1,f2**) Case 6: PDA. (**g1,g2**) Case 7: CoA. (**h1,h2**) Case 8: VSD. ccTGA: congenital corrected transposition of the great arteries; DORV: double outlet right ventricle; WS: Williams syndrome; CAF: coronary artery fistula; TOF: tetralogy of Fallot; PDA: patent ductus arteriosus; CoA: coarctation of the aorta; VSD: ventricular septal defect.

Blood pool and myocardial models were 3D printed in actual size for 8 typical cases. Both parts of the segmented myocardial model were 3D printed, and the larger part of the myocardium that can present more of the cardiac anatomy is shown in the figures. Models from the eight typical cases are presented in Fig. 2. The demographic and clinical characteristics of the cases are shown in Table 1.

**Table 1:** Demographic and clinical characteristics.

Patient demographics				Echocardiography and CT reports	Time on modeling (min)		Time on 3D printing and postprocessing (hour)		Average time of the diagnosis by CT or 3D printing (min)			Cost of 3D printing ($)	
CaseNo	Age (years)	Weight (kg)	Sex	–	Blood pool	Myocardial	Blood pool	Myocardial	CT	Blood pool3D printing	Myocardial 3D printing	Blood pool model	Myocardial model
1	19	52	Male	ccTGA	16	25	13	11.5	2.8	0.9	1.8	80	70
2	38	62	Female	DORV	14	21	8	9	2.74	0.74	1.3	50	55
3	9	32	Male	WS	11	24	5	5.5	2.4	1.35	1.98	35	35
4	3	16	Male	CAF	13	22	4.5	4.5	2.53	1.08	1.65	30	32
5	2	13	Female	TOF	9	23	3.5	4.5	2.36	1.05	1.32	22	24
6	48	56	Female	PDA	6	11	3	5	1.56	0.51	0.93	45	48
7	5	18	Male	CoA	5	9	2.5	4	1.69	0.42	0.51	25	30
8	36	51	Female	VSD	8	19	7.5	6	1.91	0.48	0.35	42	45
Mean ± SD	–	–	–	–	10.25 ± 3.67	19.25 ± 5.63	5.88 ± 3.28	6.25 ± 2.46	2.25 ± 0.44	0.82 ± 0.31	1.23 ± 0.55	41.13 ± 17.3	42.38 ± 14.2

*ccTGA* congenital corrected transposition of the great arteries, *DORV* double outlet right ventricular, *WS* Williams syndrome, *CAF* coronary artery fistula, *TOF* tetralogy of Fallot, *PDA* patent ductus arteriosus, *CoA* coarctation of the aorta, *VSD* ventricular septal defect.

### Survey and subjective evaluation

Eight typical cases were classified into two groups: complex CHDs (including ccTGA, DORV, WS, CAF, and TOF) and simple CHDs (including PDA, CoA, and VSD). Several analyses were performed to explore the adaptability of different types of 3D printing to individual kinds of CHD.

### Comparison of diagnosis results before and after 3D printing were used

**Figure 3:**
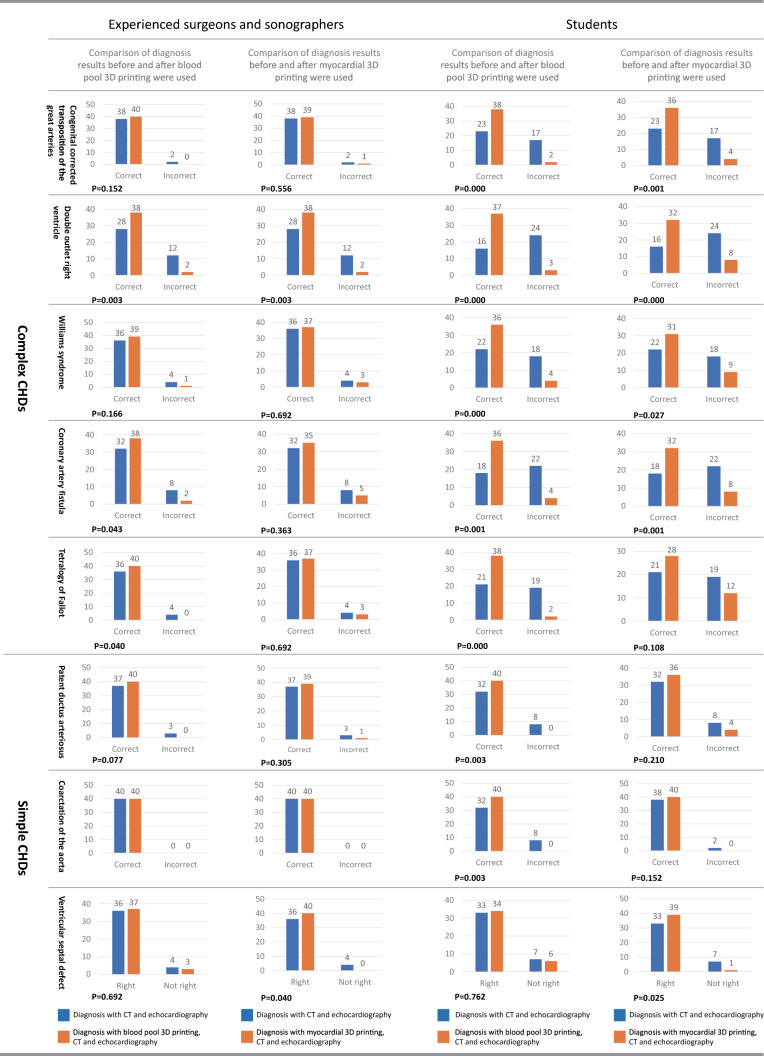
Comparison of diagnosis results before and after blood pool or myocardial 3D printing was used. The correctness of diagnosis results with CT and echocardiography, with blood pool 3D prints and echocardiography, and with myocardial 3D prints and echocardiography were compared separately.

The comparison of diagnosis results before and after 3D printing was used (Fig. 3) showed that blood pool and myocardial 3D printing have different effects on each type of CHD to varying degrees. The diagnostic impact of complex CHDs is higher than that of simple CHDs. For the expert group, the amount of change caused by 3D printing is significant mainly in complex CHDs. For the student group, 3D printing significantly improved the diagnosis of each case (*P* < 0.05).

### Comparison of diagnosis results of the expert group and student group

**Figure 4:**
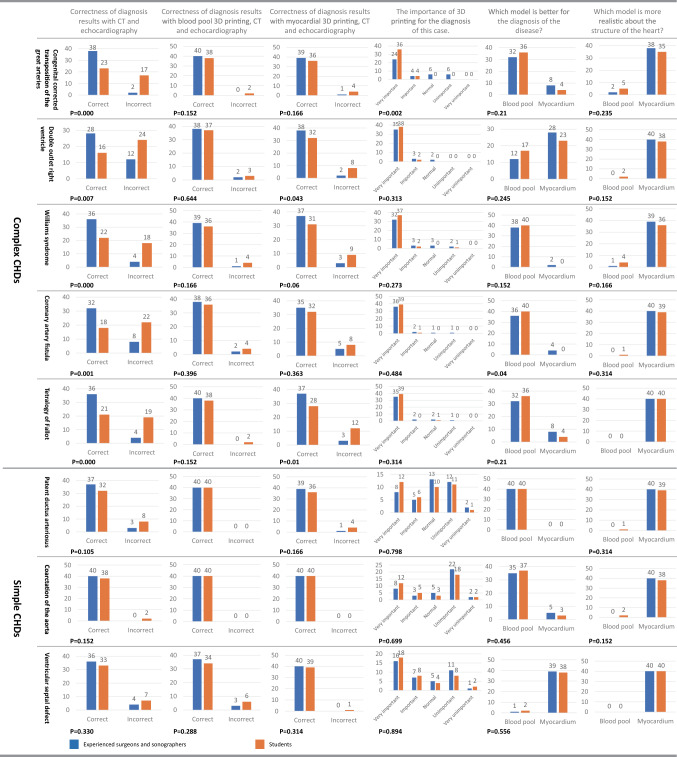
Survey results for eight typical cases between experienced surgeons and sonographers and students. The correctness of diagnosis results with CT and echocardiography, with blood pool 3D prints and echocardiography, and with myocardial 3D prints and echocardiography showed the difference of accuracy under three different diagnostic methods. The importance of 3D printing for the diagnosis of each case showed the demand for 3D printing at different levels, which showed a difference between complex CHDs and simple CHDs. The questionnaire on which model is better for the diagnosis of the disease showed the suitability of the two models for the diagnosis of each case, which differed in different cases. The questionnaire on which model is more realistic about the structure of the heart showed the similarity of two different models with heart, myocardial 3D printing is generally considered to be more similar to the actual heart.

The comparison of the expert group and student group (Fig. 4) showed that the diagnostic accuracy for complex CHDs with CT and echocardiography between the expert group and the student group was significantly different (*P* < 0.05), and the opposite was true for simple CHDs (*P* > 0.05). The average diagnostic rate before and after 3D printing was used in the 8 cases was 88.75% and 95.9% (*P* = 0.001) in the expert group and 60% and 91.6% (*P* = 0.000) in the student group, respectively. In the subjective survey of the importance of 3D printing for the diagnosis of CHDs, it was considered more important for complex CHDs than simple CHDs, and the demand of students was stronger than that of experts. In the survey of which model was better for the diagnosis of each case, most respondents selected the blood pool models (average, 74.1%) since blood pool models could clearly show the spatial relationship of cardiac structures, facilitating rapid understanding of the disease. In the case of DORV and VSD, myocardial models were considered better, as they could more effectively show the location and structure of the VSD; additionally, the blood pool model did not show the location of the VSD very well. In the survey of the realism of the structure of the heart, most of the surgeons selected the myocardial models (average, 92.5%) because it was consistent with the perspective of doctors.

### Comparison of diagnosis results of cardiac surgeons and sonographers

**Figure 5:**
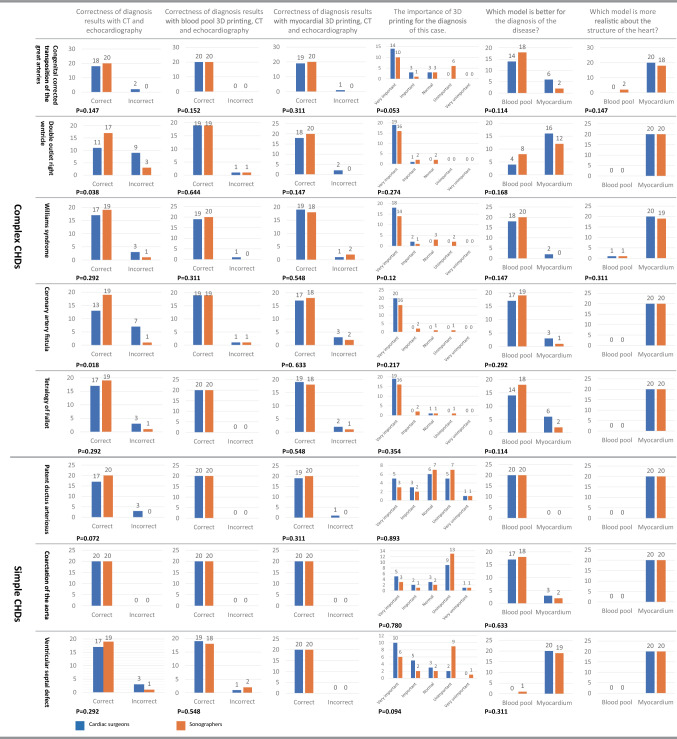
Subgroup analysis between experienced cardiac surgeons and sonographers. Blue represents the cardiac surgeon group, and red represents the sonographer group. The correctness of diagnosis results with CT and echocardiography, with blood pool 3D prints and echocardiography, and with myocardial 3D prints and echocardiography showed the difference of accuracy under three different diagnostic methods, it showed that 3D printing improved the diagnosis slightly. In the survey about the importance of 3D printing, the cardiac surgeons think more than sonographers that 3D printing is important for the diagnosis of CHDs. The importance of 3D printing for the diagnosis of each case showed the demand for 3D printing at different levels, which showed a difference between complex CHDs and simple CHDs. In the survey of which model is better for the diagnosis of the disease, the cardiac surgeon group showed a preference for myocardial models than the sonographer group.

A subgroup analysis between experienced cardiac surgeons and sonographers was carried out(Fig. 5). Overall, there was no significant difference between the two groups. The average diagnostic accuracy of cardiac surgeons(81.25%) was lower than that of sonographers(95.6%) with CT and echocardiography (*P* = 0.000). When 3D printing was used, the accuracy of correlation has been improved. The average diagnostic accuracy had risen to 97.5% for both cardiac surgeons and sonographers when blood pool 3D prints with CT and echocardiography were used for diagnosis. When myocardial 3D printing with CT and echocardiography were used, the corresponding data become 90.6% for cardiac surgeons and 96.25% for sonographers (*P* = 0.042). In the survey about the importance of 3D printing, 78.75%(very important or important) of the cardiac surgeons and 60.63%(very important or important) of the sonographers (*P* = 0.000) consider that 3D printing is important for the diagnosis of CHDs. In the survey of which model is better for the diagnosis of the disease, the overall proportion for blood pool or myocardial 3D prints on each case has not changed, but the cardiac surgeon group showed a preference for myocardial models over the sonographer group.

## Discussion

Congenital heart disease (CHD) is a type of birth defect that involves structural anomalies in the heart and major blood vessels^20^. The forms of CHD are diverse, including, but not limited to, double-outlet right ventricle (DORV), tetralogy of Fallot (ToF), ventricular septal defect (VSD), atrial septal defect (ASD), truncus arteriosus, single ventricle, etc^21^. It is crucial but difficult to diagnose types of CHD accurately. The current modalities used for diagnosis and treatment planning are computed tomography(CT), magnetic resonance imaging (MRI), and echocardiography^22^. 2D Echocardiography gives the physiological assessment of CHD and the dynamic effect on pressures or gradients. However, it can assess one defect at a time and cannot give global assessments like 3D Models. 3D Echocardiography is a great way to visualize the intracavity anatomy. It has not improved the diagnostic accuracy across the spectrum of anatomical defects due to the limited availability of acoustic windows in different subjects^23^. 3D printing provides more possibilities for the physician to further vividly understand the cardiovascular structure. Various types of 3D printing technology have been used to fabricate cardiovascular models, and we have been exploring an economic, universally accessible type of technology for this purpose. To the best of our knowledge, FDM and SLA are the most common types of 3D printing technology. Considering the speed of FDM and the limitation of support removal, SLA is the most suitable technology for manufacturing rigid cardiovascular models. Rigid blood pool and myocardial models can be made by SLA, but their effectiveness in different types of CHD has not been evaluated. In this paper, we conducted a multicase study evaluating the application of SLA 3D printing for the first time.

### 3D printing improves the diagnostic accuracy

The improvement of 3D printing over CT and echocardiography in the diagnosis of CHDs was obvious, and it was equally effective for complex and simple CHDs; in contrast, in the diagnosis of CHDs based on CT, the accuracy was lower for complex than simple CHDs. However, when 3D-printed models were used for the diagnosis, the accuracy in the student group was significantly improved, becoming similar to that in the expert group, while the diagnosis rate in the expert group also increased. 3D printing improved the accuracy of CHD diagnosis, especially among students, and this effect was more obvious for complex CHDs. The enhancement of the diagnostic accuracy is mainly due to that 3D printing improved the understanding of the cardiac structure, which is the biggest obstacle for the learning clinicians in the accurate diagnosis of CHDs. In the investigation of the necessity of 3D-printed models for the diagnosis and treatment of CHDs, most respondents considered it necessary, although we were informed by a few experts that they could identify the diseases accurately relying on their rich experience. It is undeniable that experienced experts can correctly diagnose most CHDs using echocardiography and CT. However, we have to admit that the distribution of these experts is extremely uneven, and their experience is based on extensive case training. For most doctors or students, 3D printing is necessary. Both blood pool models and myocardial models improve the diagnostic accuracy, although they have different effects in different cases.

### Blood pool and myocardial models play different roles in the diagnosis of types of CHD

**Figure 6:**
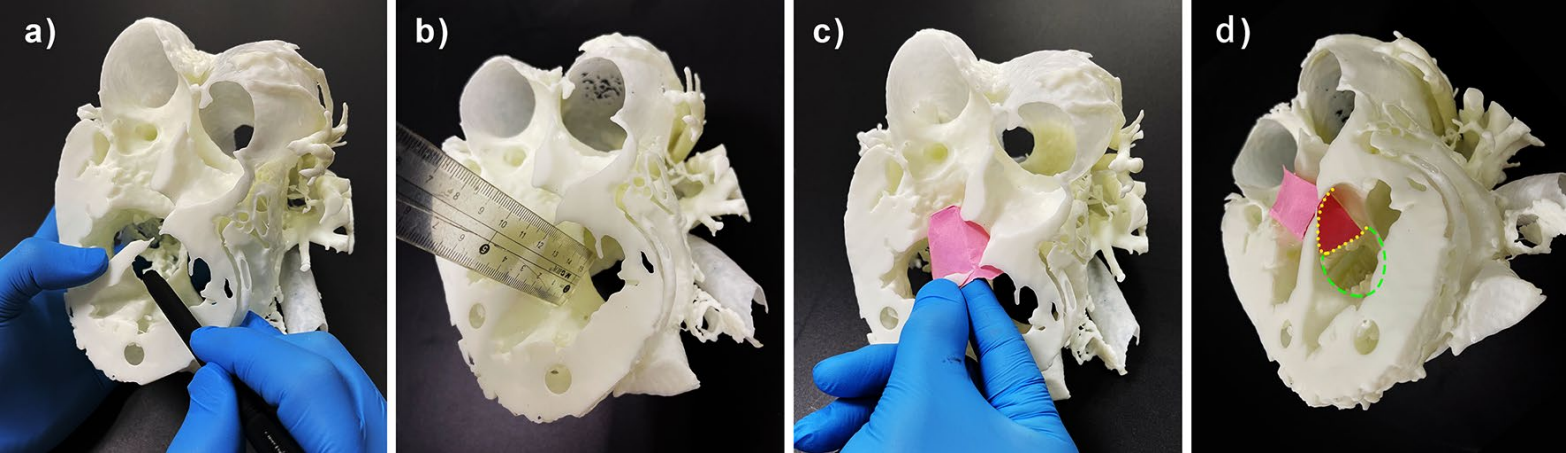
Study of the operation plan using the myocardial model in the case of DORV. (**a**) Exploration of intracardiac structures and the location of the VSD. (**b**) Direct measurement of key dimensions of the VSD. (**c**) Establishment of an internal channel from the VSD to the root of the aorta. (**d**) Simulation and presentation of the surgical plan. Yellow area: location of the intraventricular tunnel patch. Green area: location of the patch for the VSD. DORV: double outlet right ventricle; VSD: ventricular septal defect.

In cases of ccTGA, DORV, WS, CAF, and TOF, blood pool models improved the diagnostic accuracy more than myocardial models. In the research on which of the two models is more suitable for different types of CHD, the results showed a difference. In cases of ccTGA, WS, CAF, TOF, PDA, and CoA, i.e., CHDs with “structural heterotopia”, blood pool models were considered to be more effective, as they were good at illustrating arteriovenous connections, vessel stenosis/obstruction, and chamber volumes. However, the results were the opposite in cases of VSD and DORV. In the case of VSD (Fig. 2h1), the location of the VSD was occluded by the left and right ventricles, so it was not easy to find. In the myocardial model (Fig. 2h2), the VSD was shown as a hole, which was easy to find and understand. Similarly, in the case of DORV (Fig. 2b1), the blood pool model was useful for finding the origin of the root of the aorta and the pulmonary artery. However, when we performed in-depth research, the surgical plan and myocardial model (Fig. 2b2) were found to be more important, as they helped doctors accurately estimate the exact location of the VSD (Fig. 6a), the relationship of the VSD to the septal leaflet of the tricuspid valve, the subaortic or subpulmonary outflow tract, and the distance between the upper margin of the VSD and the nearest arterial valve (Fig. 6b). In addition, the model allowed the doctor to simulate channel establishment (Fig. 6c,d) and estimate the volume of the remaining right ventricle(RV) after application. The myocardial model can better illustrate the ventricular structure, which will benefit the diagnosis of CHDs judged refer to the ventricular, especially the RV structure, such as DORV, VSD, and TOF, etc.

### Myocardial models are more similar to the actual heart than blood pool models

From the aspect of shape similarity, the myocardial model is more similar to the actual heart than the blood pool model. However, this does not mean that the myocardial model is the best choice for the diagnosis of all cases. For the cases of ccTGA, WS, CAF, TOF, PDA, and CoA, blood pool models are preferred. In more in-depth interviews, some respondents stated that the combination of the two could be better for the diagnosis of the disease. Although there were no significant differences, the experienced doctors generally preferred the myocardial models to the blood pool models. This is probably because they see more hearts in the first view, while the students are trained to establish the spatial relationship of various parts in the heart cavity, which is more similar to the blood pool models. This may have something to do with our habit of understanding the structure of the heart. Usually, each cavity, such as the left and right ventricles, is considered as an entity, which is the same as the structure of the blood pool. This could be why the students preferred the blood pool models. This habit gradually changed after they looked at the heart from the first perspective for a long time.

### Both surgeons and sonographers need 3D printing for diagnosis

The subgroup analysis between experienced cardiac surgeons and sonographers showed a slight difference between the two groups. The diagnostic accuracy of surgeons is slightly lower than that of sonographers in the traditional diagnosis with CT and echocardiography, the application of 3D printing narrowed the gap. The survey about the importance of 3D printing confirmed the view that 3D printing is more important for cardiac surgeons than sonographers. This may be due to the fact that sonographers are specialized in and better at diagnosis, and in the process of investigation, echocardiography has always been one of the methods used for diagnosis. But with 3D printing, cardiac surgeons can also make better diagnoses. In addition, another survey result indicated that the cardiac surgeon group showed a preference for myocardial models over the sonographer group. This may be related to the habit of understanding the heart: sonographers doctors are more concerned about hemodynamics, while cardiac surgeons are more concerned about the structure of the myocardium.

### Time and cost on different modeling and 3D printing

Compared with colorful blood pool models applied for the diagnosis of CHDs^4,22^, monochrome blood pool models have a disadvantage at first glance, but they do not affect the accuracy of the diagnosis. In terms of surgical simulation, flexible myocardial models can better train doctors for surgery, while hard myocardial models have shortcomings in this regard. However, considering the cost of the models, this deficiency can be ignored. A study on the time and price of modeling, 3D printing, and postprocessing showed that the average cost of 3D printing of blood pool and myocardial models was approximately 41.8 dollars, which is much less expensive than printing multicolor models or soft models^24^. The average time of modeling for the blood pool and myocardial models was approximately 10 min and 20 min, respectively, and the average time of 3D printing and postprocessing was within 7 h, which allows large-scale application. It is worth mentioning that the time and cost of blood pool models and myocardial models are slightly different. The average time on blood pool modeling is minutes shorter than that on myocardial modeling, but the difference is negligible compared to a few hours on 3D printing. The cost of myocardial models is higher than that of blood pool models due to the influence form the structure of myocardial models. Therefore, the blood pool models are superior to the myocardial models in cost.

Research on the average time of diagnosis using CT or 3D printing has shown that 3D printing allows a diagnosis to be made faster. The diagnosis of CHDs mainly depends on the judgment of the cardiac structures. When diagnosing using CT, 3D spatial relationships are produced through planar images, which is a very difficult and time-consuming process because it requires the comparison of almost every slice. However, 3D printing establishes and displays these spatial relationships, leaving only a judgment to be made based on the visible 3D model. In some cases, only one glance is needed to find the location and condition of the lesion, such as in cases of VSD, PDA, and CoA. Generally speaking, there are some differences in time and cost on modeling, 3D printing and postprocessing, and preferences for specific applications on blood pool models and myocardium models.

### Comparison of virtual models and 3D printing

In addition to improving diagnostic accuracy, 3D printing is of great significance in clinical decisions of the treatment strategy. 3D printing established the assessment of anatomical defects, which is of great utility for the correct identification of anatomy. Besides, the individualized complex structure for complex CHDs can enhance the understanding of 3D Echocardiography and provide a method for hemodynamic simulation and evaluation, which improved the understanding of the pathophysiological impact on flow, pressures, and gradients. Furthermore, 3D printing provides a physical object for measuring, evaluating, and simulating the surgery, which helps surgeons make accurate surgical plans.

Before 3D printing, the virtual model created can also be used for the diagnosis of CHDs. The advantages and disadvantages of augmented reality, mixed reality, virtual reality, and 3D printing have been compared^25^. On the whole, virtual models have the advantages of fast, low-cost, and repeatable application, but this method also requires more skills from the operator. In addition, the virtual models and 3D printing could be cut at any angle. The display of the internal structure of the heart is crucial for diagnosis. Although cardiac CT with 3D reconstruction has been used in the diagnosis of CHDs^26,27^, it can show the outer surface of the heart model, there are deficiencies in showing the internal structure of the myocardial model. The advantage of 3D printing lies in the physical characteristics of the model and high quality of the simulation. The perception of spatial relationships will be biased on a virtual screen, but the 3D printing of objects can eliminate this bias because the objects can be touched as if they were on a real operating table, and all the perceptions and simulations of the physical model can then be applied to the real heart.

### Limitations

Several limitations of this study must be noted. First, only one case of each CHD was selected in the comparative study, it is difficult to generalize the diagnostic accuracy on specific heart disease with n = 1. While each CHD usually includes a wide spectrum of anatomical variations. Study of large sample size should be studied in subsequent studies. Second, in the questionnaire survey on the diagnostic accuracy of CT, blood pool 3D printing, and myocardial 3D printing, echocardiography has always been used as an auxiliary way, which may affect the results of the survey. A more detailed survey of each technology individually or in combination can be performed.

## Conclusion

The use of rigid 3D-printed models can improve the diagnosis of CHDs, and this improvement is more obvious for complex CHDs. Blood pool models and myocardial models had different effects on improving the diagnostic accuracy in different cases. In cases of ccTGA, WS, CAF, TOF, PDA, and CoA, which are characterized as CHDs with “structural heterotopia”, blood pool models were more effective; in cases of VSD and DORV, myocardial models showed more advantages in showing the structure of the lesion. The model should be selected with consideration of the category of CHD in practical application.

## Supplementary Information

Supplementary Information.

## Data Availability

All data are available on request at the Department of Cardiovascular Surgery, Peking University International Hospital, No.1, Zhongguancun Life Science Park, Beijing, China.
