# Supplementary material for: Comparison of blood pool and myocardial 3D printing in the diagnosis of types of congenital heart disease
Source: Sci Rep. 2022 May 3;12:7136. doi: 10.1038/s41598-022-11294-6 (PMC9065034; doi:10.1038/s41598-022-11294-6)
Supplement: Supplementary file 1 — Supplementary Information. [file 41598_2022_11294_MOESM1_ESM.docx]

**Detailed comparison of the individual kinds of CHD**

The demographic and clinical characteristics of the cases are shown in Table 1. In order to better demonstrate the impact of 3D printing on diagnosis, we show the diagnosis details of each case here.

| Case 1 | Congenital corrected transposition of the great arteries (ccTGA)+ Pulmonary hypertension (PH) |
| --- | --- |
| Echocardiography | 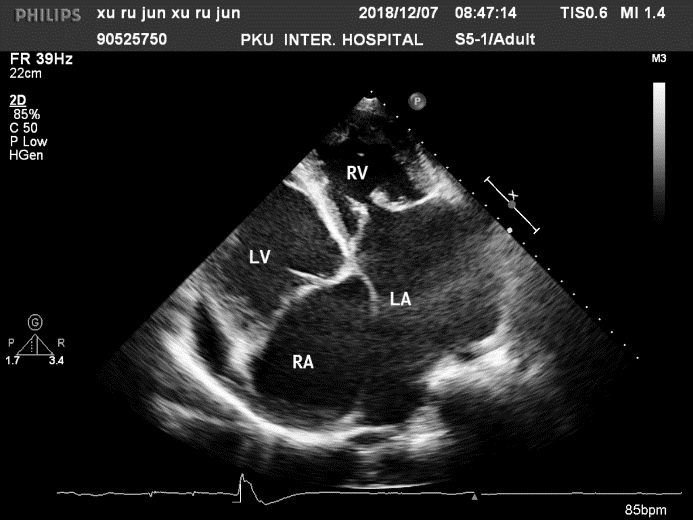 |
| CT image | 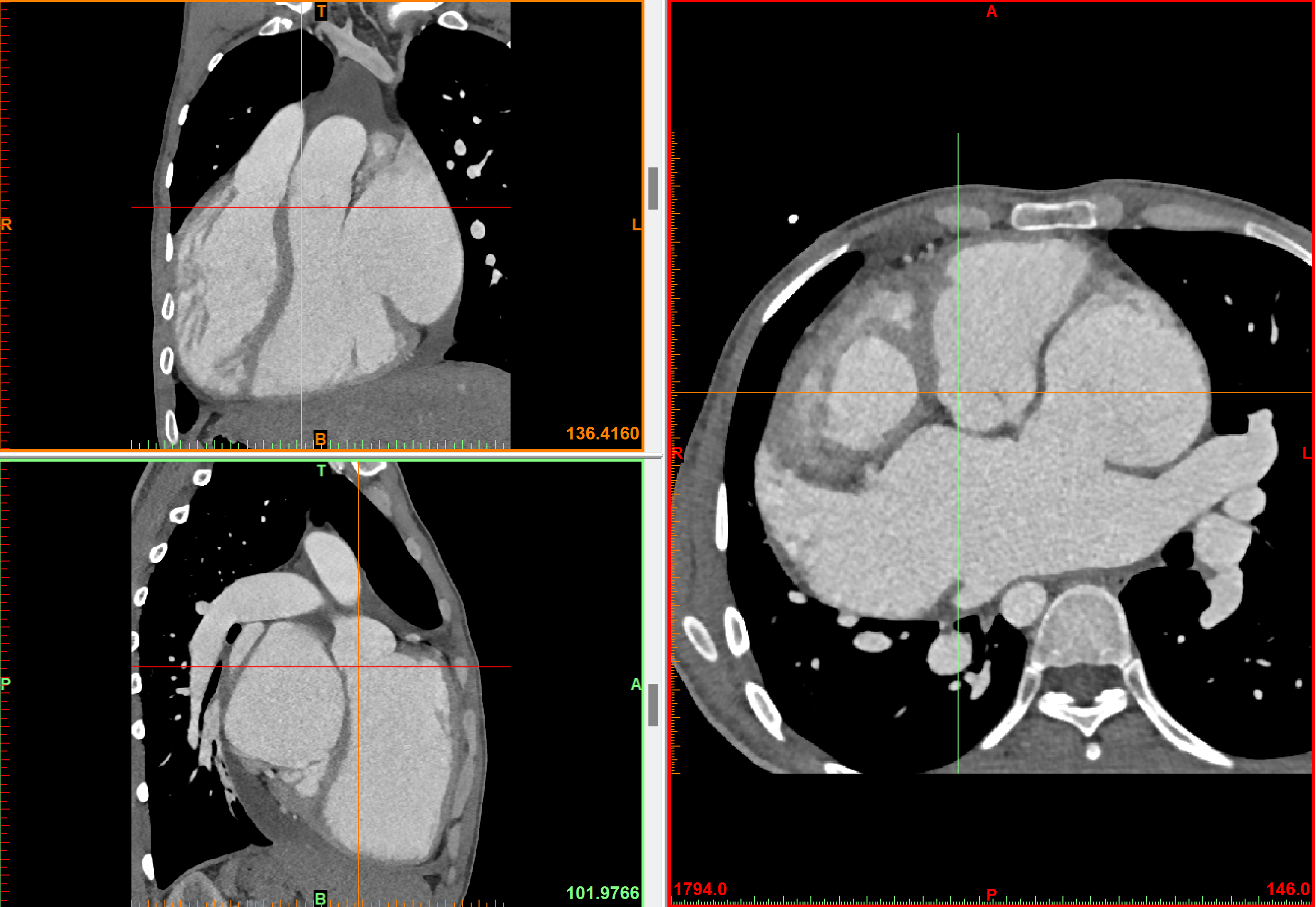 |
| Blood pool 3D printing | 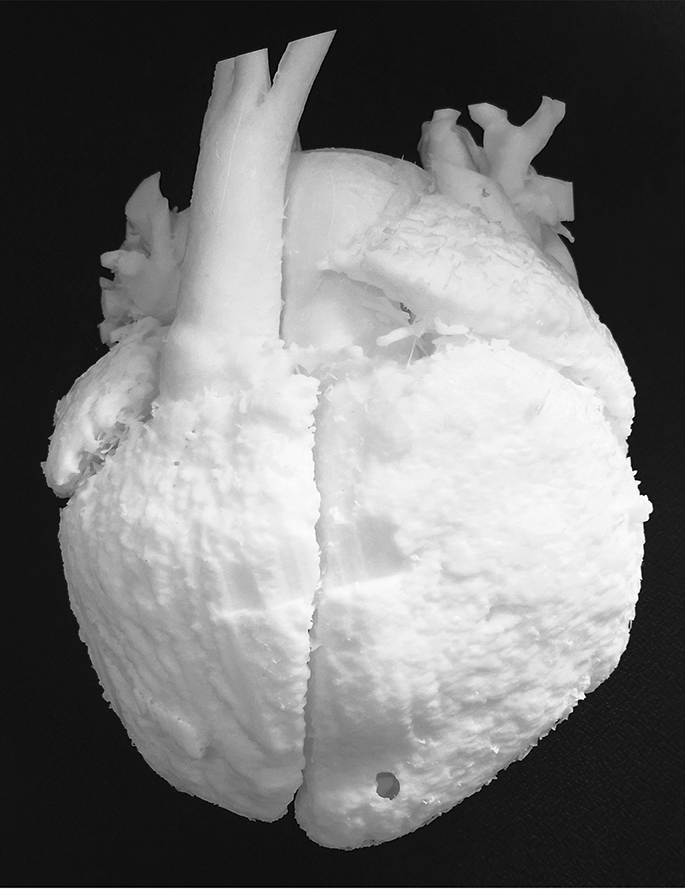 |
| Myocardial 3D printing | 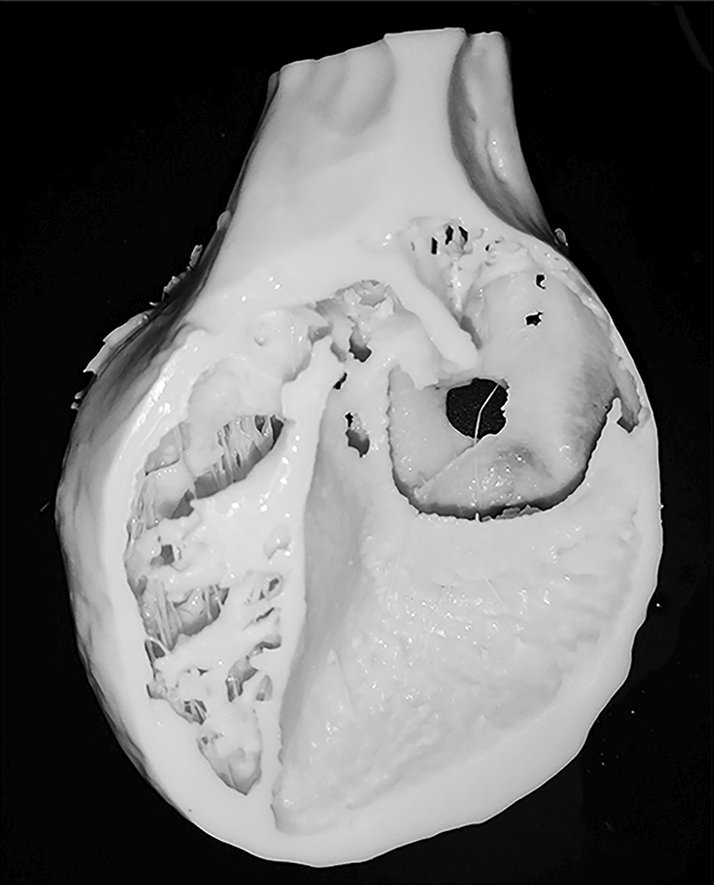 |
| Problems in traditional diagnosis methods and the reasons | As shown in the CT image, the anatomical structure and spatial relationship cannot be displayed directly. It is necessary to rely on the professional knowledge and spatial imagination of surgical response to restore the internal structure, which is prone to errors. It was wrongly diagnosed as complete transposition of great arteries in CT diagnosis. It is easy to be missed when diagnosed using echocardiography. |
| Improvement of 3D printing on diagnosis and the reasons | 3D printing plays an important role in the diagnosis of this case. The location and size of pulmonary artery(PA) and aorta(Ao) are obvious, the left ventricle(LV) and right ventricle(RV) are easy to distinguish because of the clear papillary muscle structure. The blood pool model shows the relationship between Ao, Pa, LV and RV better than myocardial model. The myocardial model better shows the thickness of the myocardium and its comparison with the blood pool. But in general, because the blood pool model has more advantages in showing the various regions in the heart and their positional relationship, the blood pool model is more conducive to the diagnosis of this case. |

| Case 2 | Double outlet right ventricle (DORV)+ Pulmonary hypertension (PH) |
| --- | --- |
| Echocardiography | 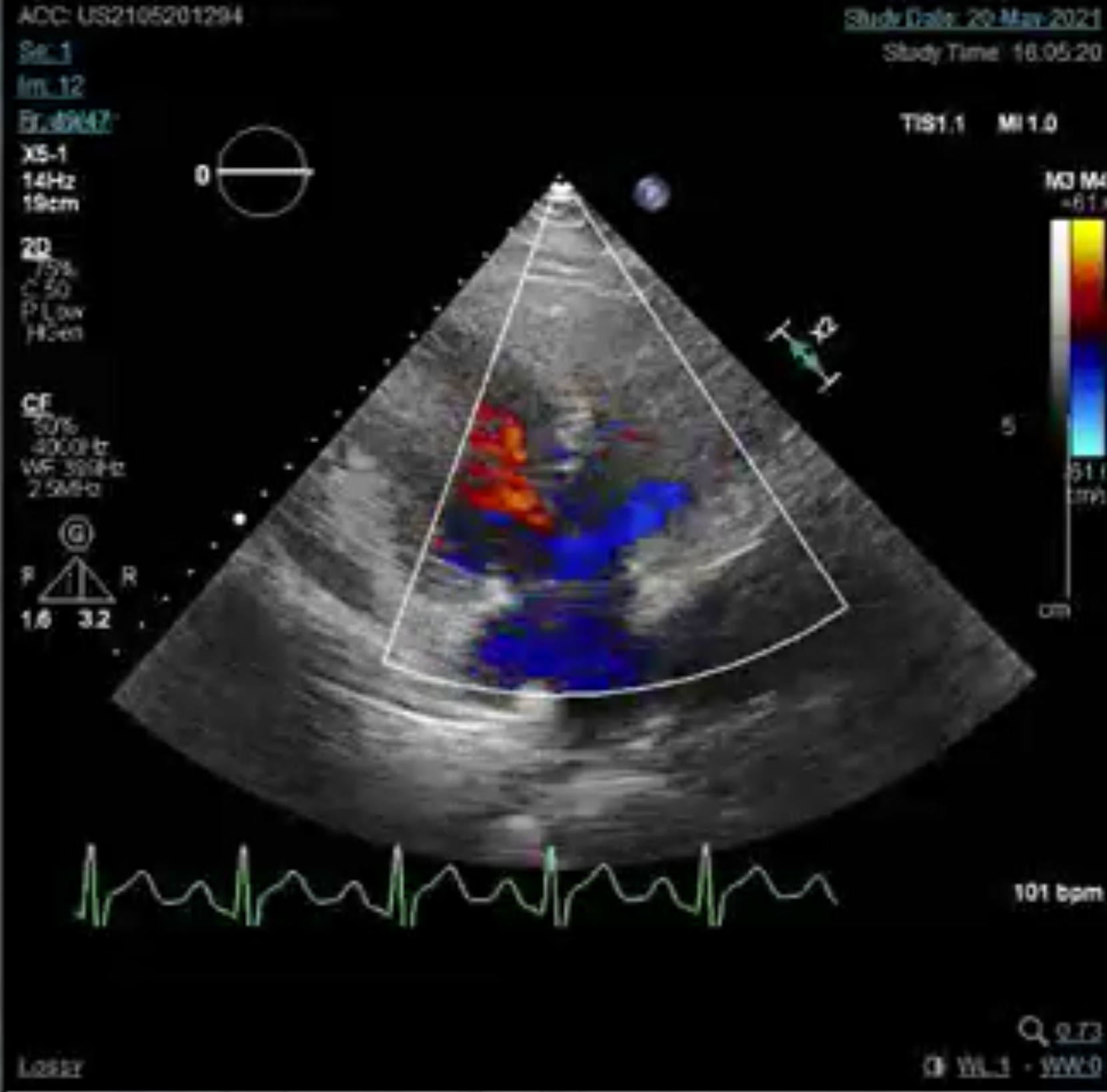 |
| CT image | 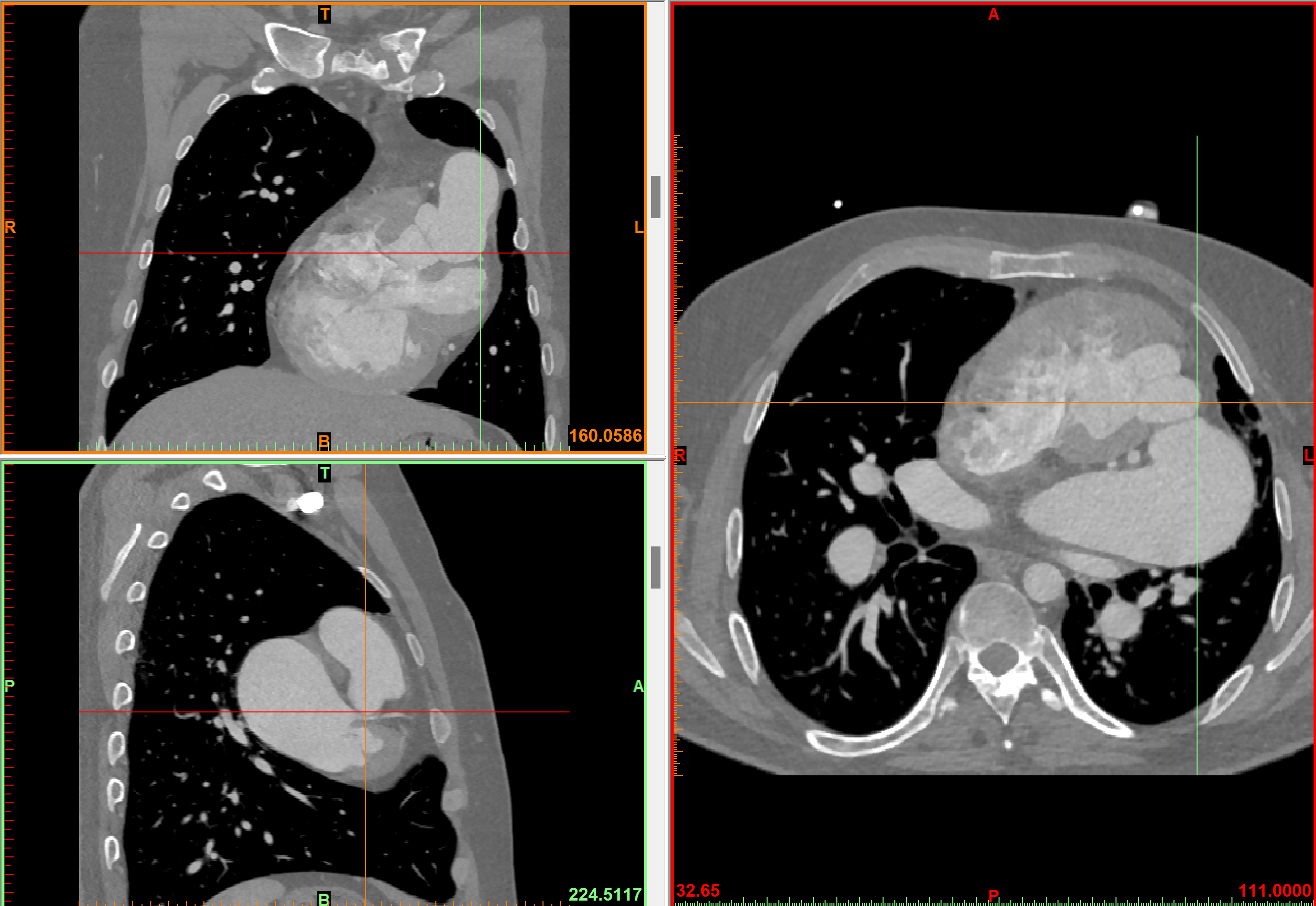 |
| Blood pool 3D printing | 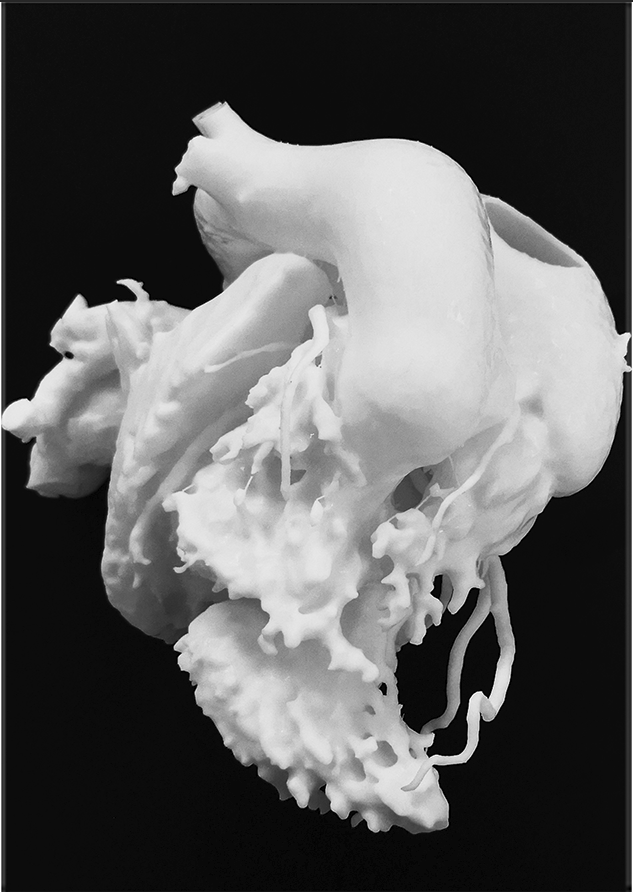 |
| Myocardial 3D printing | 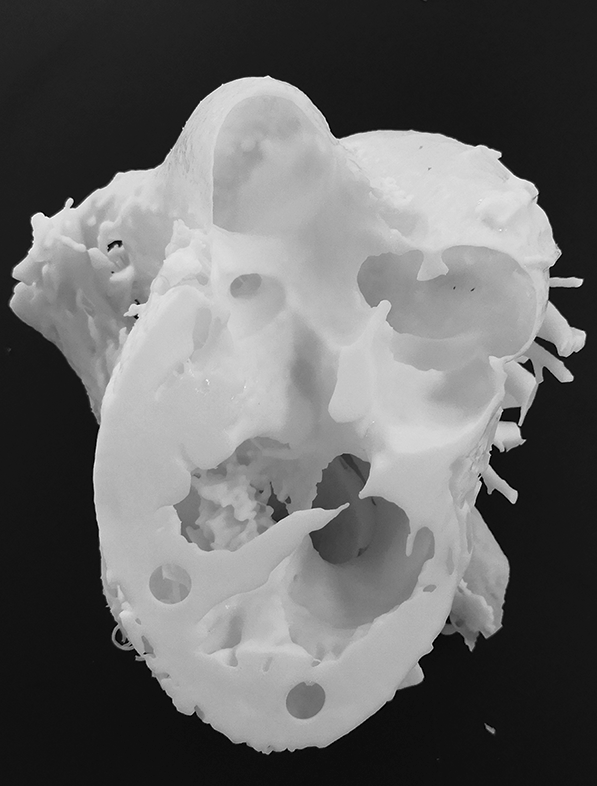 |
| Problems in traditional diagnosis methods and the reasons | The diagnosis of DORV is difficult in traditional ways. As shown in the CT image, the angle and position of aortic offset, the location and size of ventricular septal defect(VSD) are difficult to visualize and quantify. In the diagnostic statistics based on echocardiography and CT, it was misdiagnosed as overriding Ao and VSD. |
| Improvement of 3D printing on diagnosis and the reasons | Blood pool and myocardial 3D printing clearly show the location of the Ao, PA and VSD. It is obvious that both the Ao and the PA originate from RV. In the in-depth study of diagnosis and surgical scheme, myocardial model can be used to accurately measure the diameter of the VSD, and observe the distance and angle between the Ao and the VSD. Myocardial model is more important in the diagnosis of this case, and has more advantages in simulating and evaluating the feasibility of establishing inner tunnel from VSD to Ao. |

| Case 3 | Williams syndrome |
| --- | --- |
| Echocardiography | 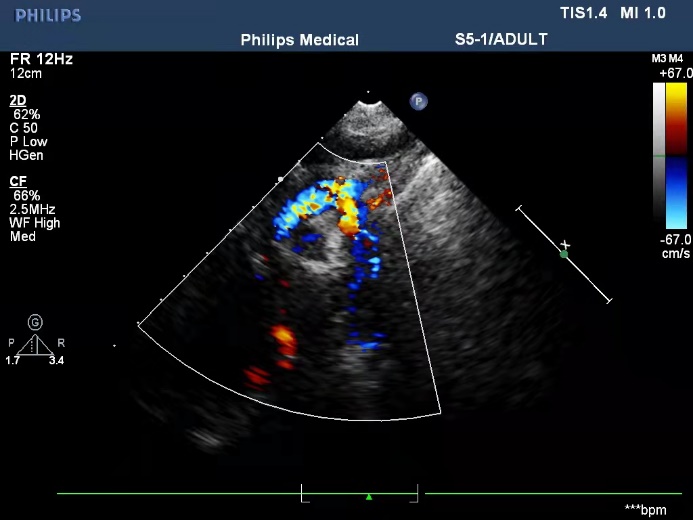 |
| CT image | 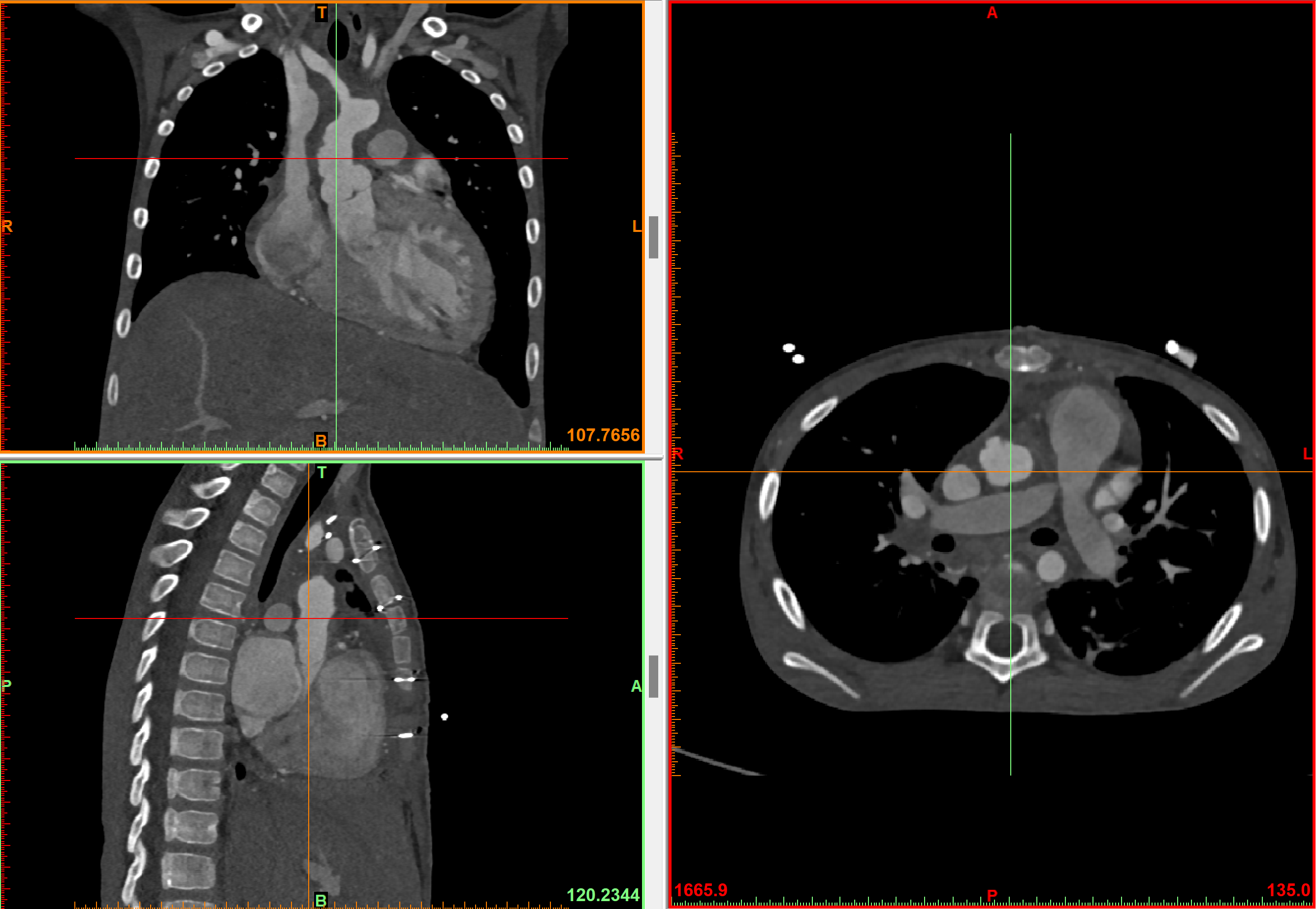 |
| Blood pool 3D printing | 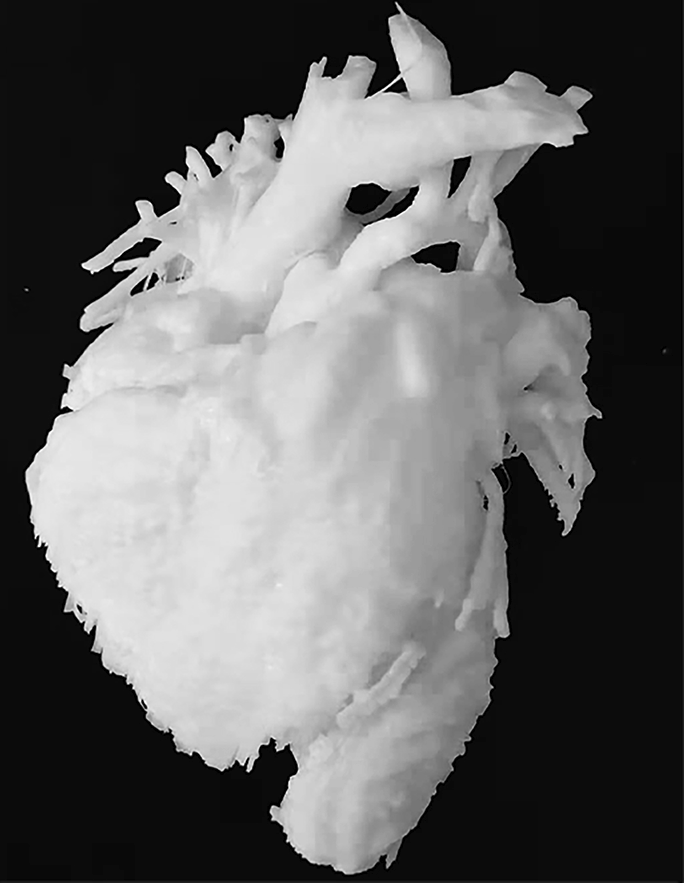 |
| Myocardial 3D printing | 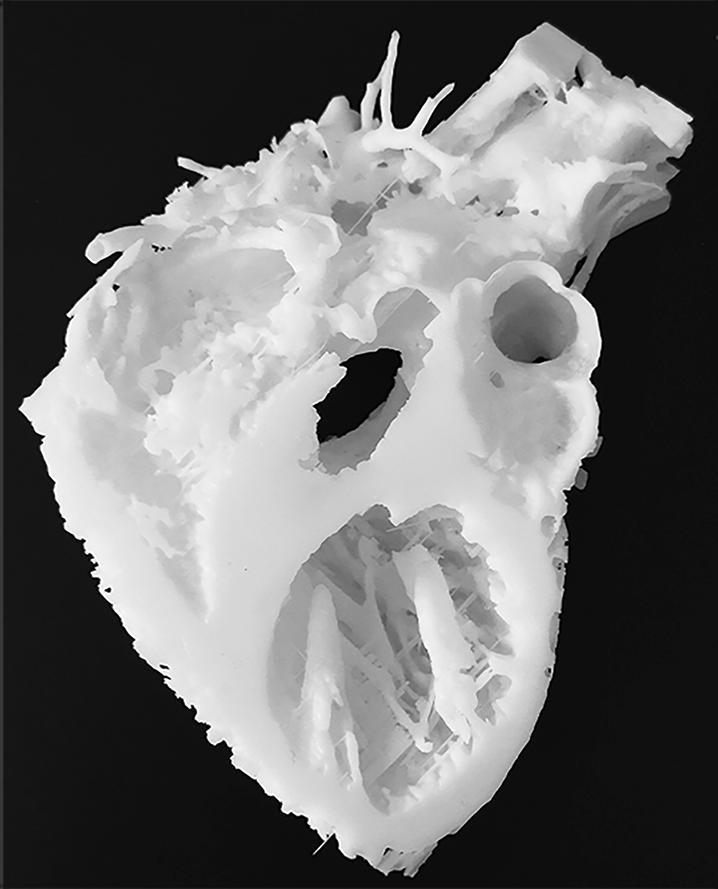 |
| Problems in traditional diagnosis methods and the reasons | The traditional diagnosis of Williams syndrome mainly depends on ultrasonography, which needs to consider the stenosis of Ao and PA at the same time, so it is easy to miss diagnosis. Some investigators misdiagnosed it as aortic stenosis and right brachiocephalic aneurysm by CT and ultrasonography. |
| Improvement of 3D printing on diagnosis and the reasons | Blood pool 3D printing plays an important role in the diagnosis of this case. The blood pool model clearly showes the size changes of Ao and PA. It is easy to see multiple aortic stenosis and right brachiocephalic trunk aneurysm. However, in the myocardial model, the cavity structure of Ao and PA is easy to be blocked. Therefore, the blood pool model can better improve the diagnosis of this case. |

| Case 4 | Coronary artery fistula (CAF) |
| --- | --- |
| Echocardiography | 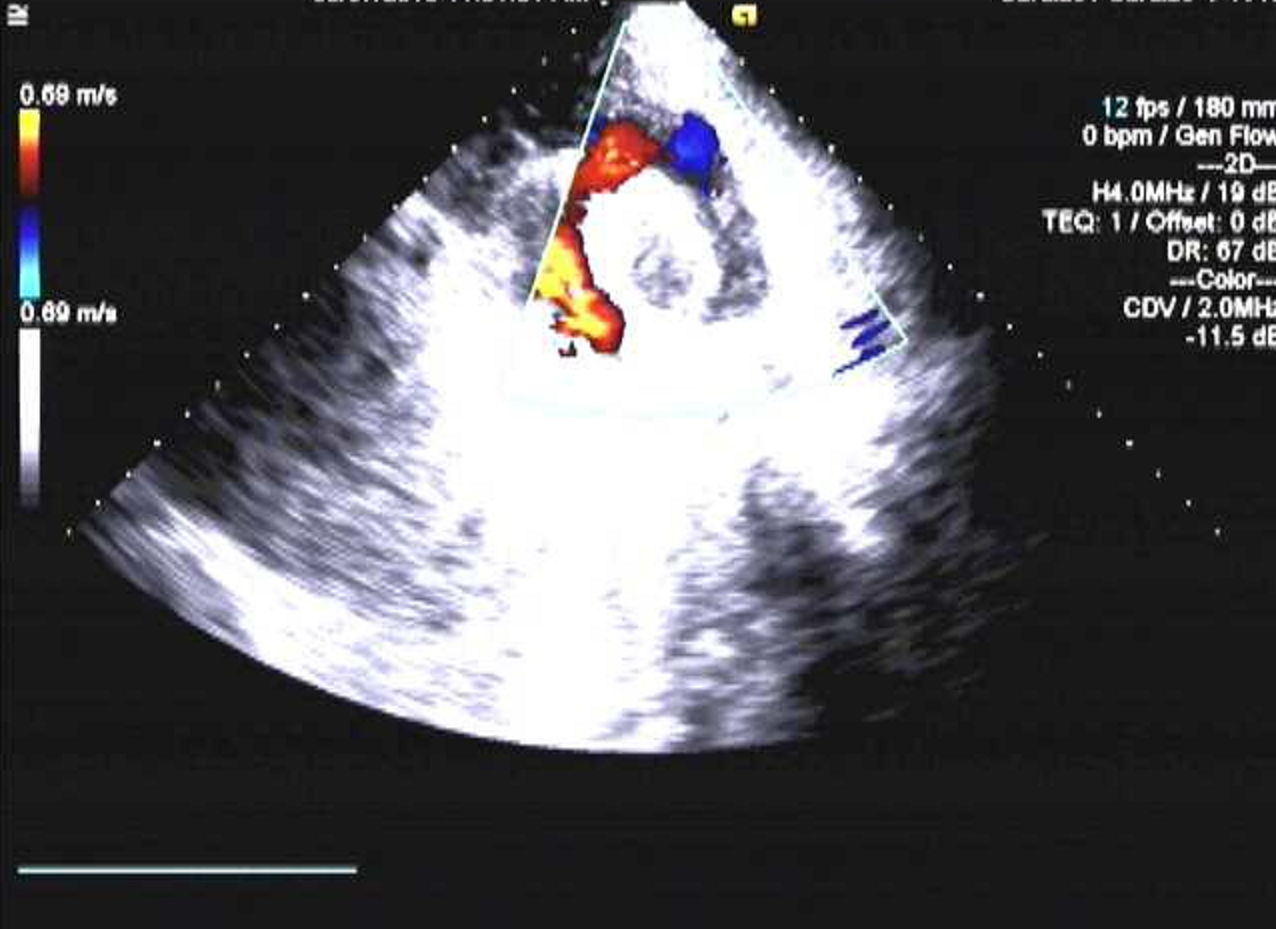 |
| CT image | 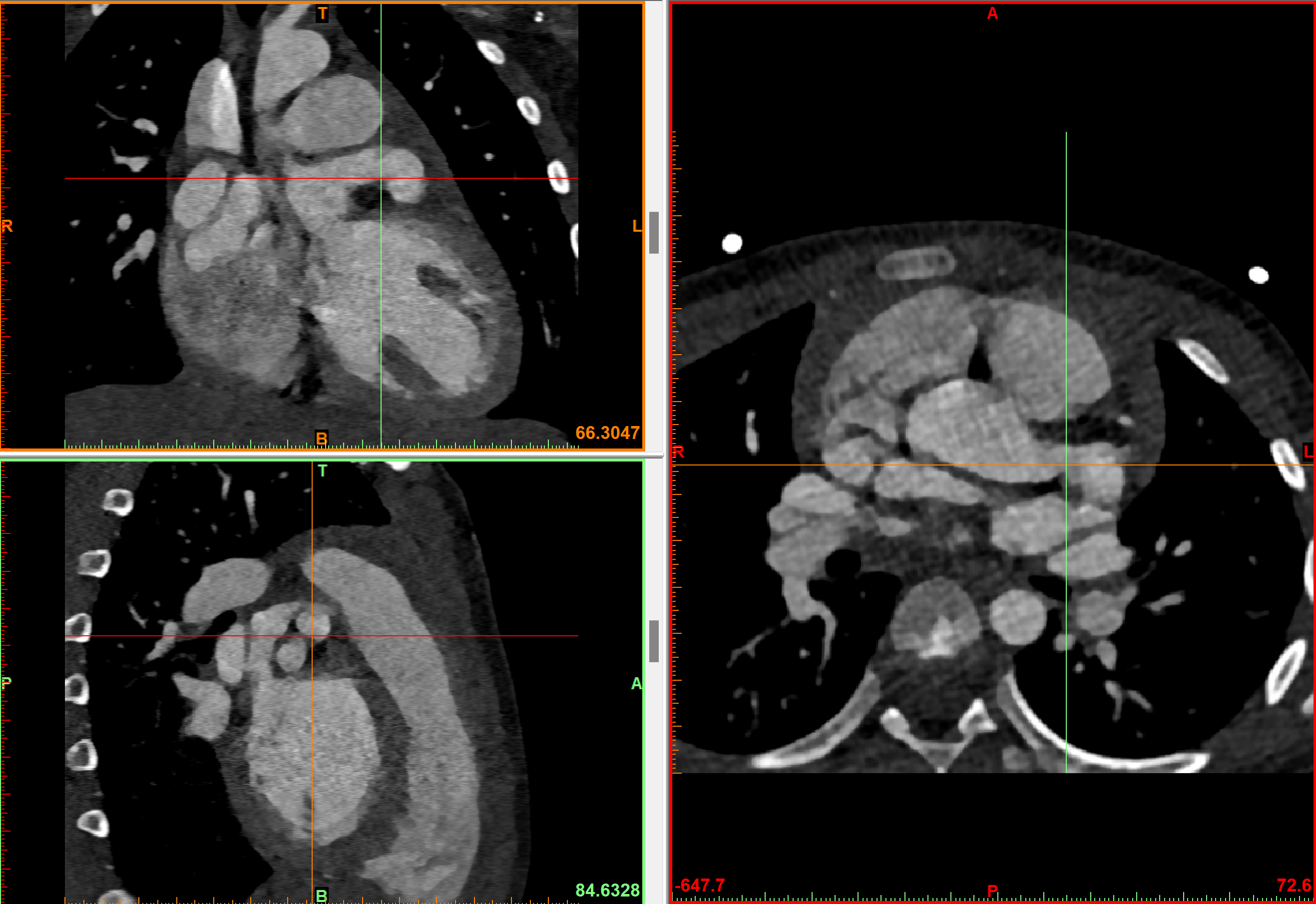 |
| Blood pool 3D printing | 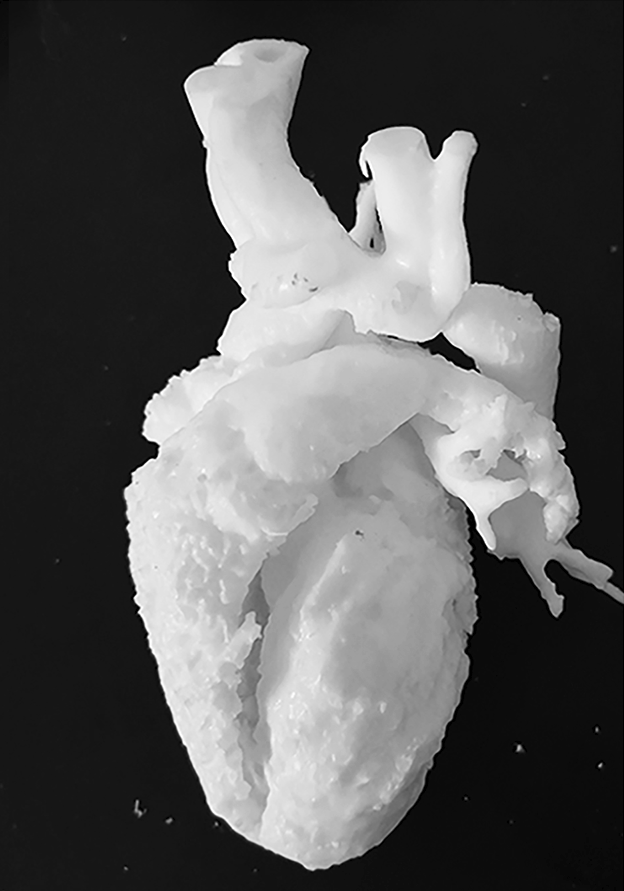 |
| Myocardial 3D printing | 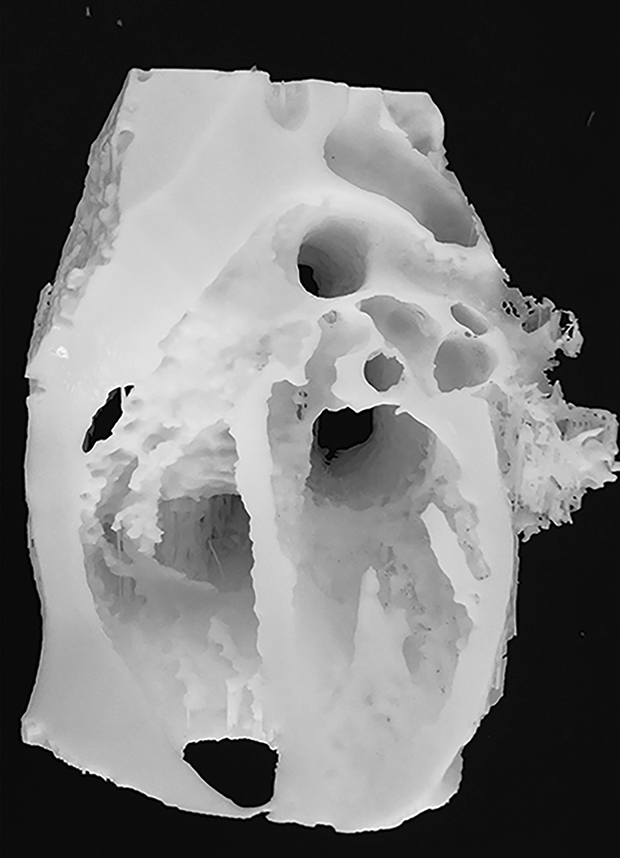 |
| Problems in traditional diagnosis methods and the reasons | It is difficult to clearly show the course and shape of coronary artery and its connection with Ventricles by CT and echocardiography. Therefore, there are some missed diagnoses in the traditional diagnosis. It was misdiagnosed as coronary right ventricular fistula by echocardiography, and misdiagnosed as circumflex branch tumor like dilatation because it is difficult to show the structure of coronary fistula by CT. |
| Improvement of 3D printing on diagnosis and the reasons | The blood pool model shows that the anatomical structure of coronary fistula, and the myocardial model shows coronary-right atrial fistula. The fistula in the right atrium can be clearly observed. Combined with 3D printing, doctors and students can better understand the complex structure, and the diagnosis rate is also greatly improved. On the whole, the blood pool model shows the internal and vascular structure of the heart more obviously, which is more helpful to the diagnosis of this case. |

| Case 5 | Tetralogy of Fallot (TOF) |
| --- | --- |
| Echocardiography | 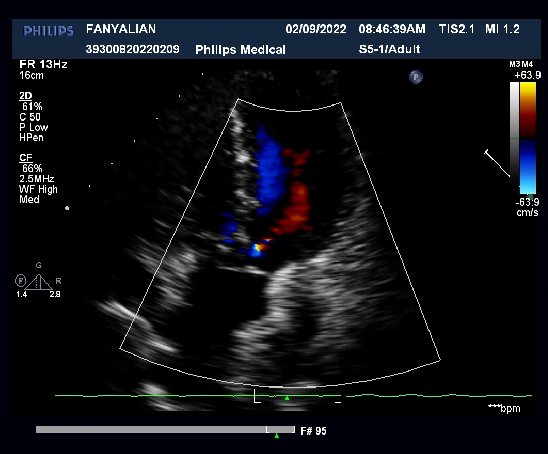 |
| CT image | 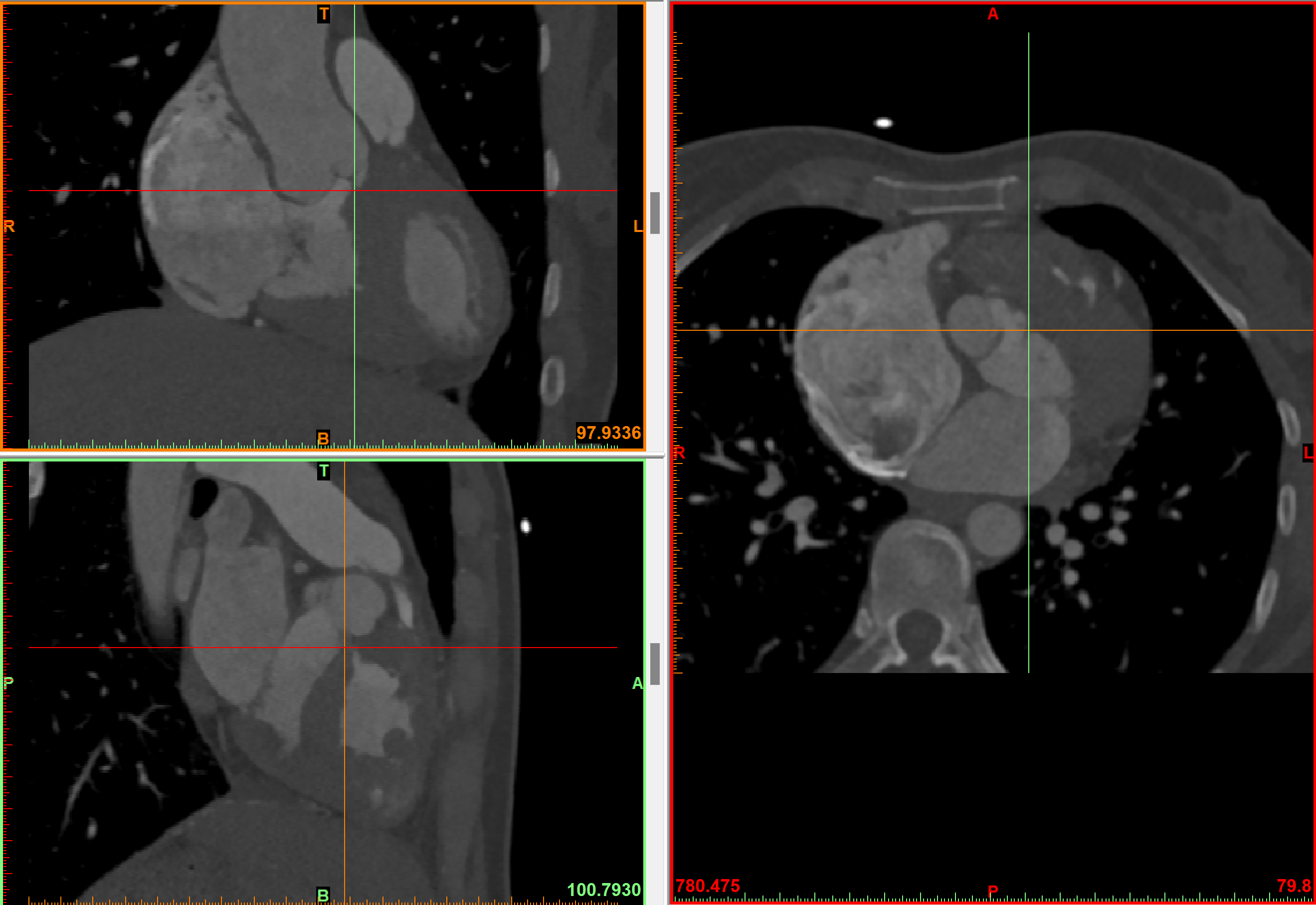 |
| Blood pool 3D printing | 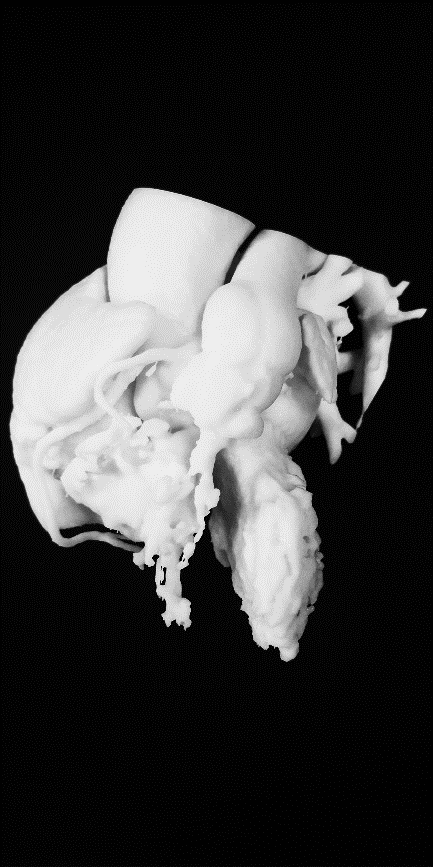 |
| Myocardial 3D printing | 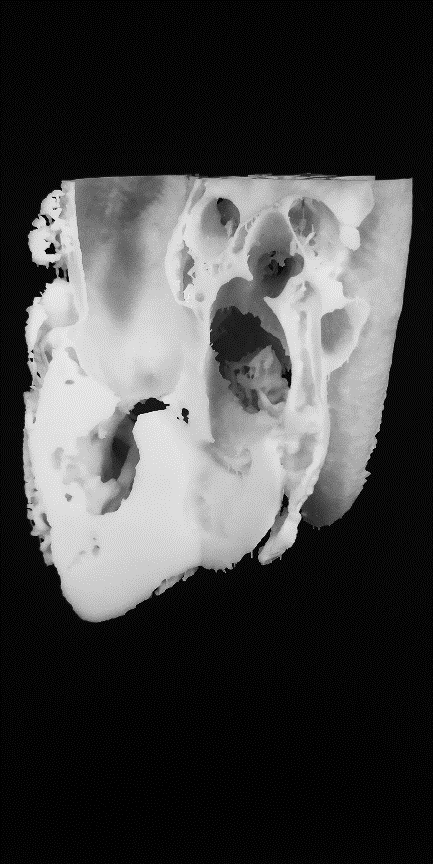 |
| Problems in traditional diagnosis methods and the reasons | The main diagnostic basis of tetralogy of Fallot(TOF) is the position of aortic straddle. It is not intuitive to observe the relative position of Ao root and VSD by CT. In the traditional diagnosis of this case, it might be misdiagnosed as VSD with aortic straddle using echocardiography, and misdiagnosed as VSD using CT. |
| Improvement of 3D printing on diagnosis and the reasons | The blood pool model can clearly show the relative position of Ao and VSD, the Ao rides over the VSD. The stenosis of right ventricular outflow tract can also be clearly observed by the blood pool model. The myocardial model shows the degree of aortic riding clearly, but not as comprehensive as the blood pool model. Combined with 3D printing, it can be clearly diagnosed as TOF. |

| Case 6 | Patent ductus arteriosus (PDA) |
| --- | --- |
| Echocardiography | 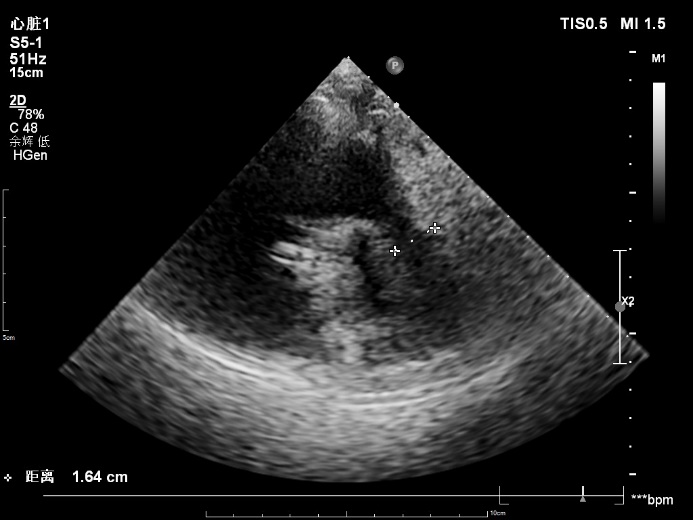 |
| CT image | 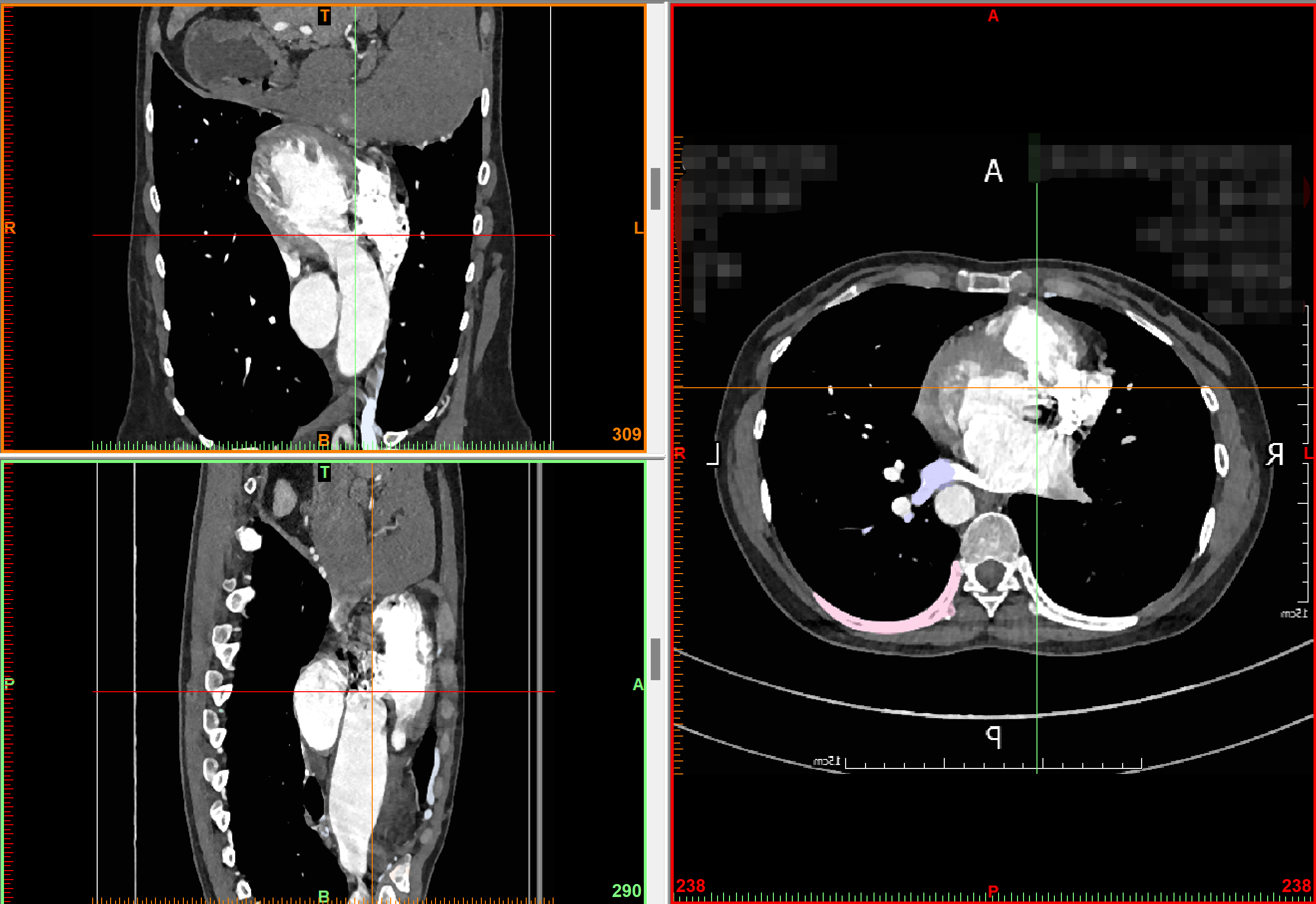 |
| Blood pool 3D printing | 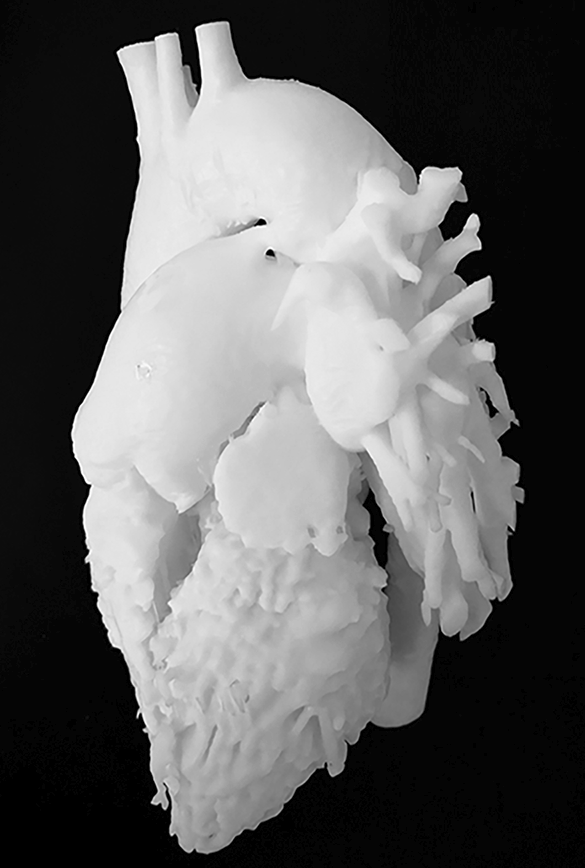 |
| Myocardial 3D printing | 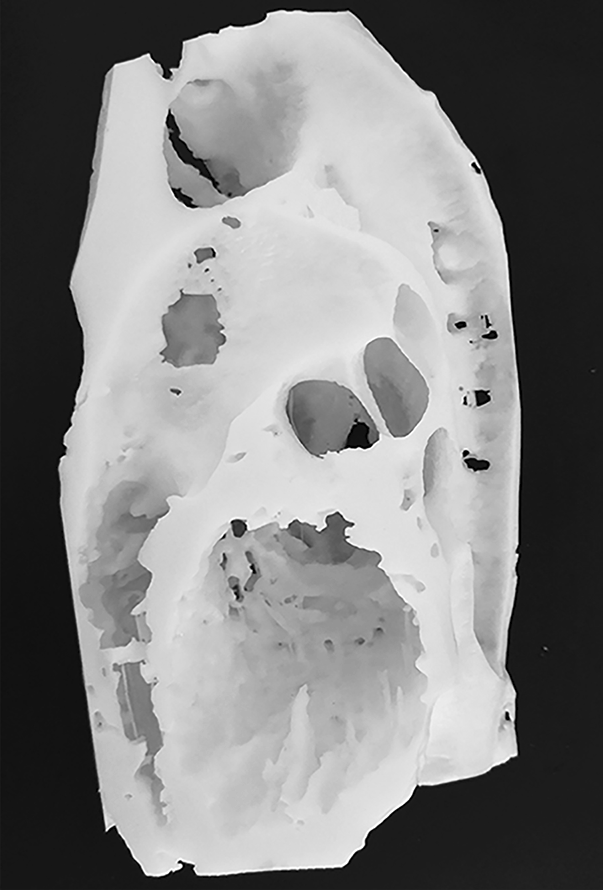 |
| Problems in traditional diagnosis methods and the reasons | Because the diameter of patent ductus arteriosus (PDA) varies greatly among individuals, small PDA is easy to be ignored when diagnosing by echocardiography and CT. There was a missed diagnosis |
| Improvement of 3D printing on diagnosis and the reasons | The arterial catheter is shown clearly by blood pool 3D printing. In the myocardial model, the position of the section may affect the display of the arterial catheter. Therefore, from the perspective of diagnosis, the blood pool model is considered to be better for this case. |

| Case7 | Coarctation of the aorta (CoA) |
| --- | --- |
| Echocardiography | 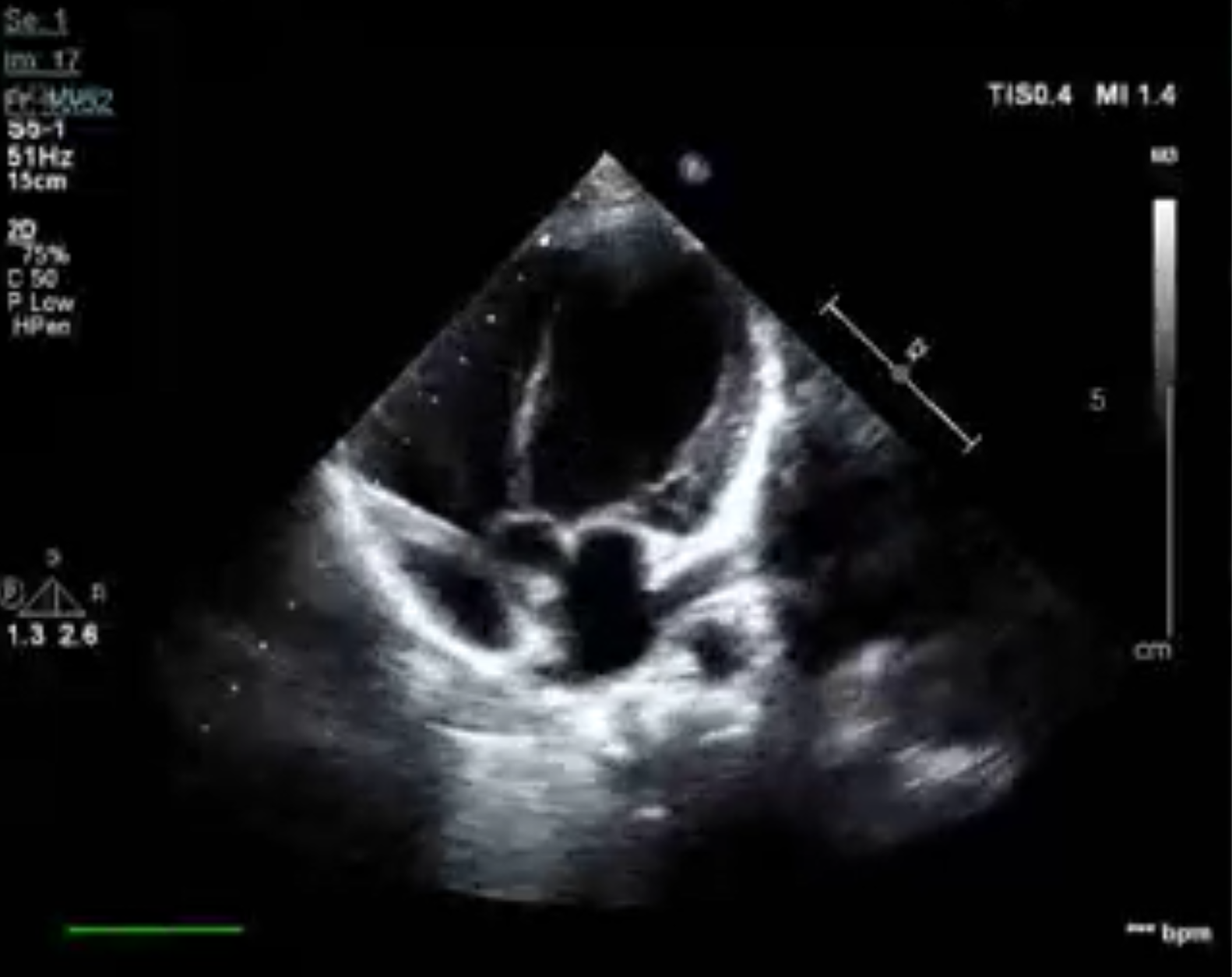 |
| CT image | 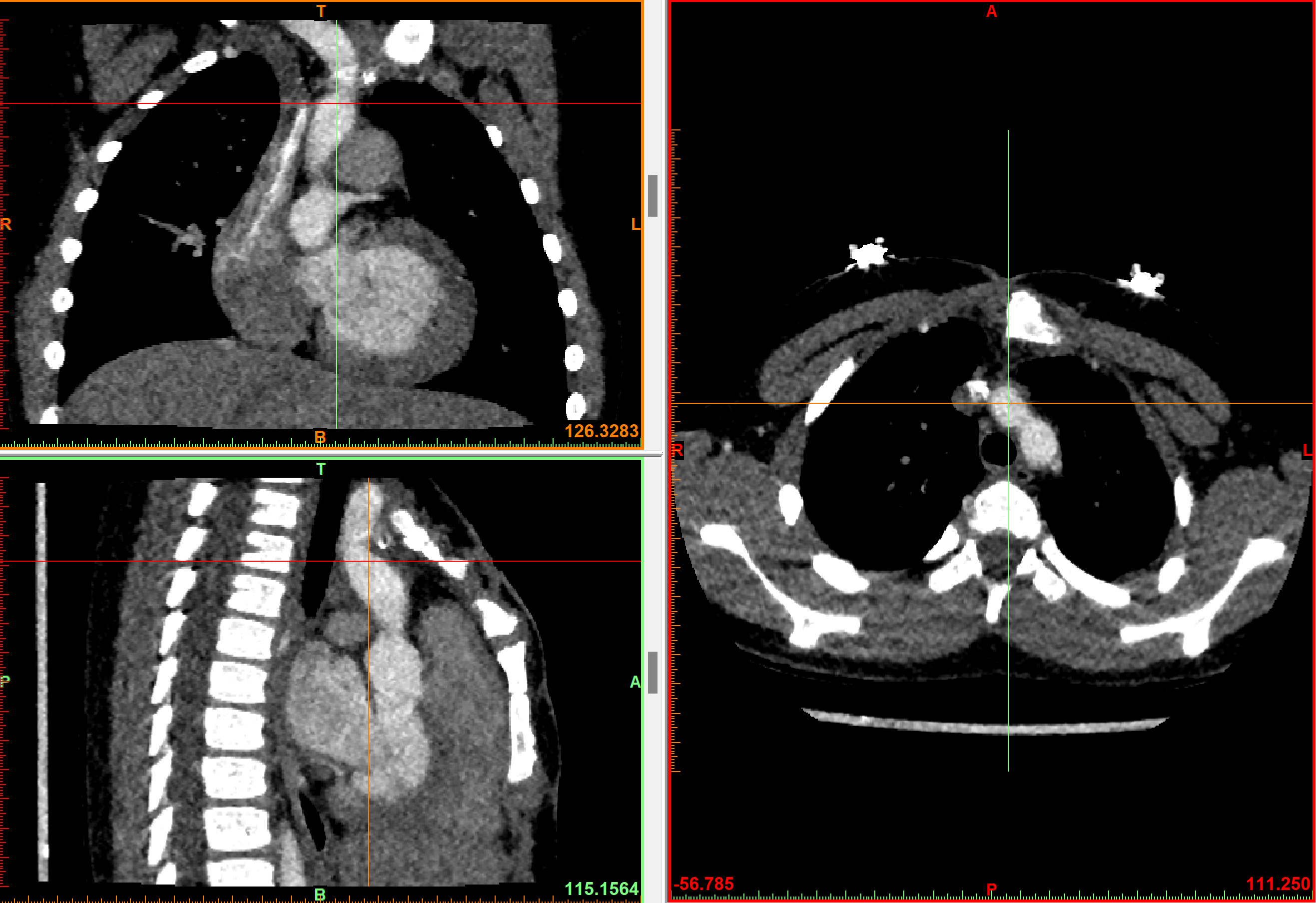 |
| Blood pool 3D printing | 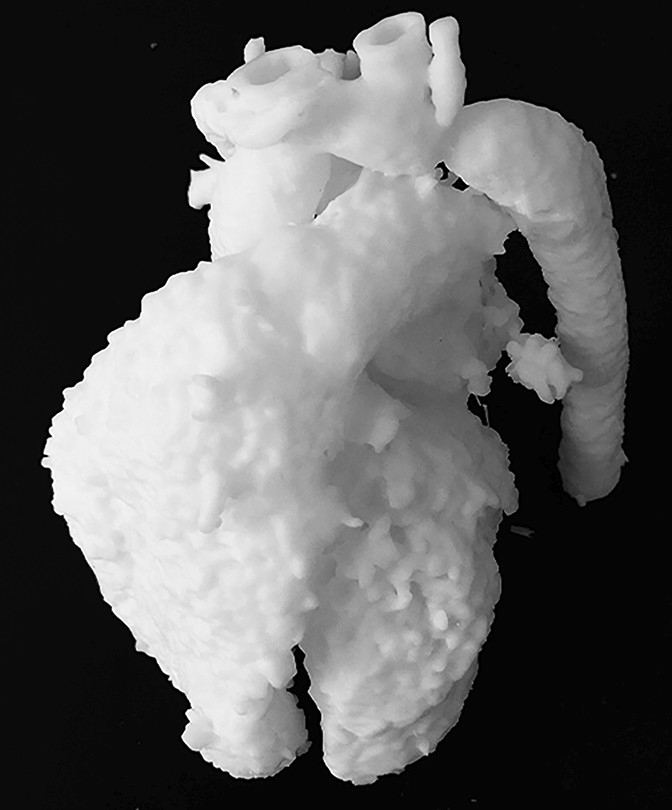 |
| Myocardial 3D printing | 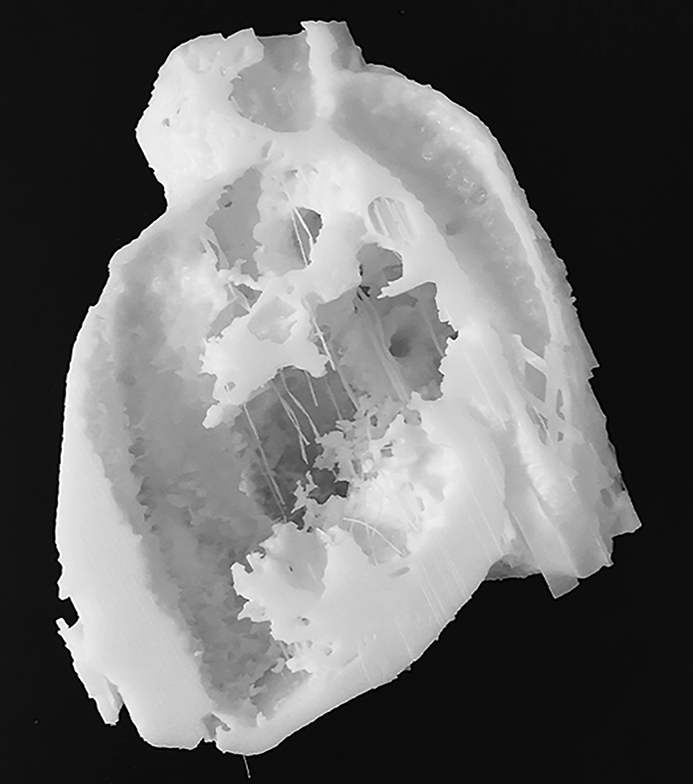 |
| Problems in traditional diagnosis methods and the reasons | As simple CHD, coarctation of the aorta (CoA) is easy to be diagnosed. The location and degree of CoA can be easily showed by echocardiography and CT. There is almost no misdiagnosis occurred in the diagnostic tests. |
| Improvement of 3D printing on diagnosis and the reasons | Both blood pool model and myocardial model can be well used for diagnosis. The blood pool model is considered better because it shows the morphology of the Ao more intuitively,. |

| Case 8 | Ventricular septal defect (VSD)+ Pulmonary hypertension (PH) |
| --- | --- |
| Echocardiography | 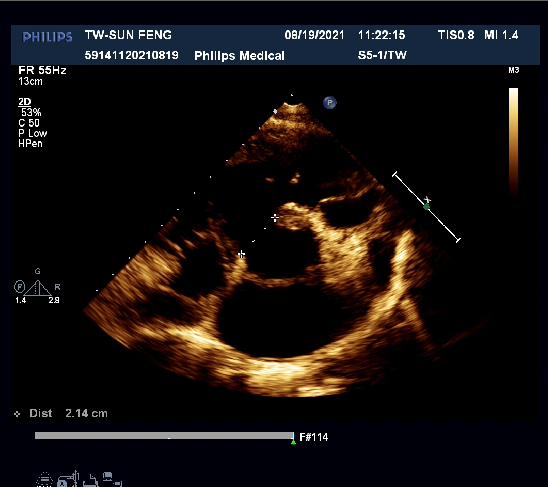 |
| CT image | 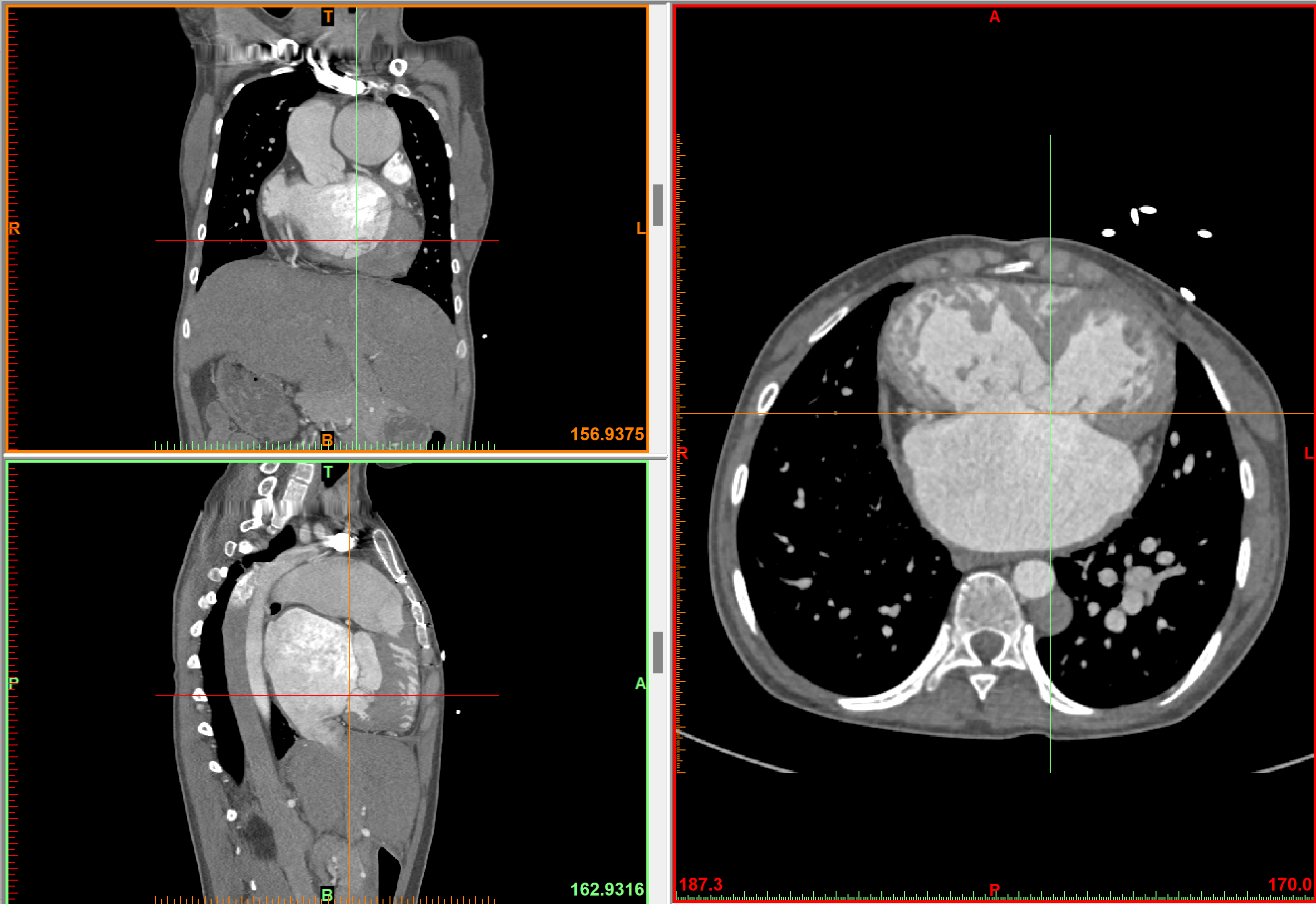 |
| Blood pool 3D printing | 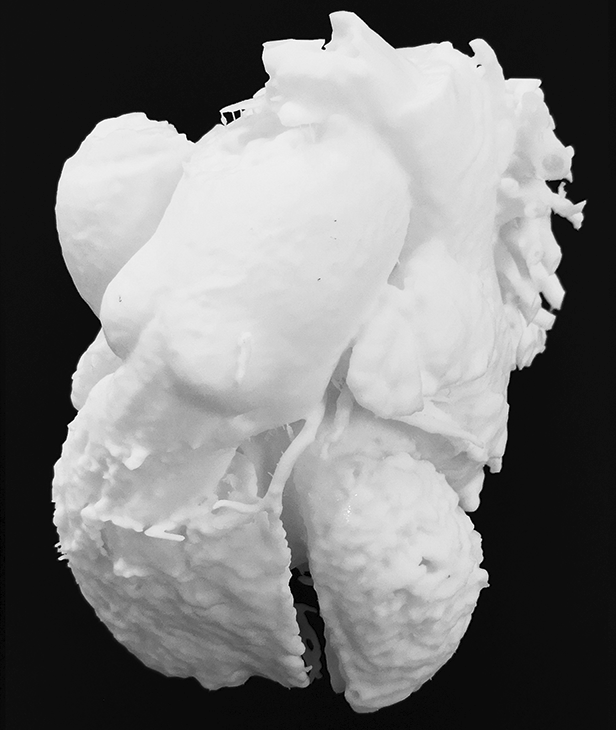 |
| Myocardial 3D printing | 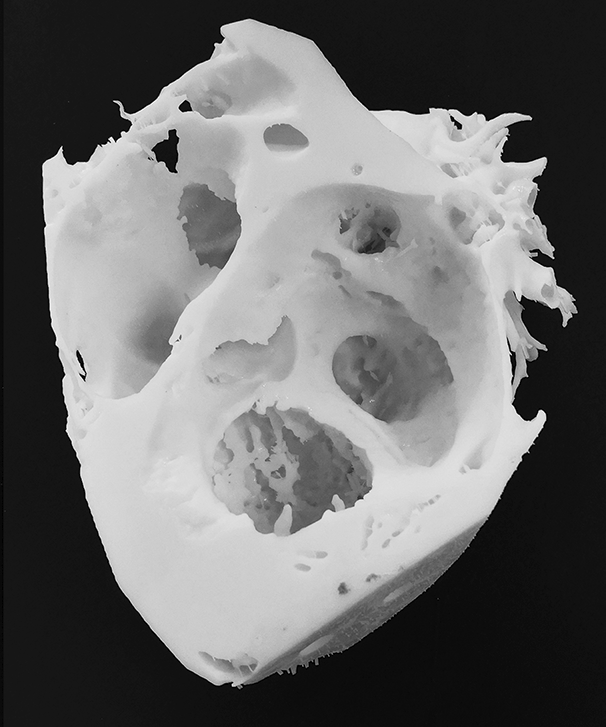 |
| Problems in traditional diagnosis methods and the reasons | As simple CHD, VSD is easy to be diagnosed. The location and degree of VSD can be easily showed by echocardiography and CT. There is almost no misdiagnosis occurred in the diagnostic tests. |
| Improvement of 3D printing on diagnosis and the reasons | The myocardial model can directly show the location of VSD, which is shown as a hole in the model obviously. In the blood pool model, the VSD is the connection between LV and RV, which can be easily blocked by the LV and RV. |
